# Immobilized cellulose nanospheres enable rapid antigen detection in lateral flow immunoassays

**DOI:** 10.1007/s10570-022-05038-y

**Published:** 2023-01-05

**Authors:** Katariina Solin, Marco Beaumont, Maryam Borghei, Hannes Orelma, Pascal Mertens, Orlando J. Rojas

**Affiliations:** 1 Department of Bioproducts and Biosystems, School of Chemical Engineering, Aalto University, Vuorimiehentie 1, 00076 Espoo, Finland; 2 VTT Technical Research Centre of Finland Ltd., Tietotie 4E, 02044 Espoo, Finland; 3 Department of Chemistry, Institute of Chemistry for Renewable Resources, University of Natural Resources and Life Sciences Vienna (BOKU), Konrad-Lorenz-Straße 24, 3430 Tulln, Austria; 4 Coris BioConcept, Rue Jean Sonet 4A, 5032 Gembloux, Belgium; 5 The Bioproducts Institute, Departments of Chemical and Biological Engineering, Chemistry and Wood Science, University of British Columbia, 2360 East Mall, Vancouver, BC V6T 1Z4 Canada

**Keywords:** Immunoassays, Cellulose nanoparticles, Paper-based diagnostics, Patterning, Protein interactions, Coronavirus antigen test

## Abstract

Rapid diagnostic systems are essential in controlling the spread of viral pathogens and efficient patient management. The available technologies for low-cost viral antigen testing have several limitations, including a lack of accuracy and sensitivity. Here, we introduce a platform based on cellulose II nanoparticles (oppositely charged NPan and NPcat) for effective control of surface protein interactions, leading to rapid and sensitive antigen tests. Passivation against non-specific adsorption and augmented immobilization of sensing antibodies is achieved by adjusting the electrostatic charge of the nanoparticles. The interactions affecting the performance of the system are investigated by microgravimetry and confocal imaging. As a proof-of-concept test, SARS-CoV-2 nucleocapsid sensing was carried out by using saliva-wicking by channels that were stencil-printed on paper. We conclude that inkjet-printed NPcat elicits strong optical signals, visible after a few minutes, opening the opportunity for cost-effective and rapid diagnostic.

**Graphical abstract:**

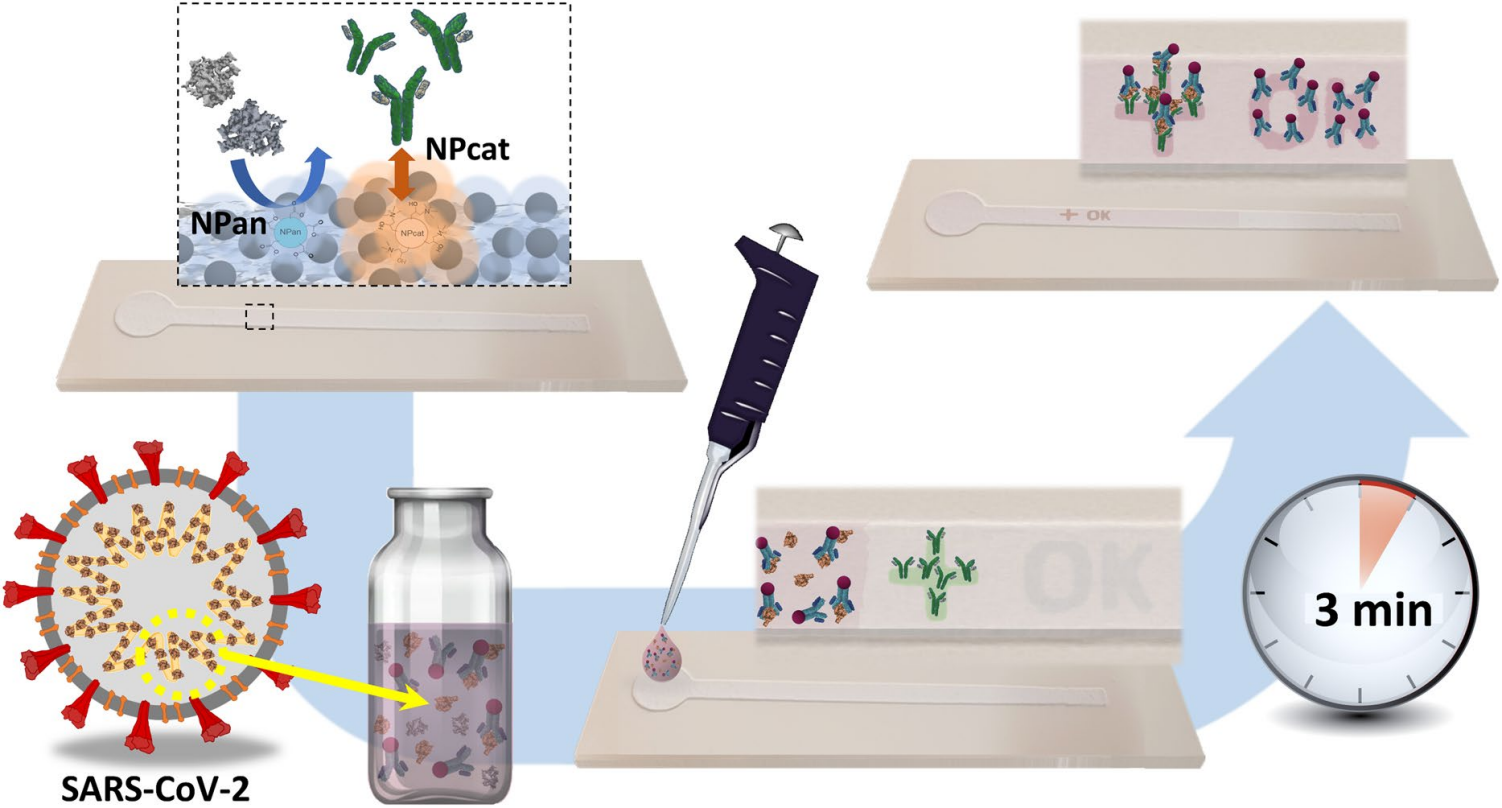

**Supplementary Information:**

The online version contains supplementary material available at 10.1007/s10570-022-05038-y.

## Introduction

The COVID-19 pandemic has accelerated research aiming to develop diagnostic systems suitable for wide-scale screening of viral infections, enabling better control of virus transmission. Currently, the most common diagnostic methods for SARS-CoV-2 infection are reverse transcription-polymerase chain reaction (RT-PCR) and immunoassays (Carter et al. 2020). The main advantage of RT-PCR is the high sensitivity, as only minor amounts of viral RNA are needed for amplification. However, PCR-based diagnosis requires skilled personnel for sampling and analysis (Feng et al. 2020). This has been a demanding aspect that limits healthcare resources and capacity.

Immunoassays are also instrumental in the diagnosis of viral infections, especially because they allow rapid point-of-care (POC) testing. Indeed, many reports describe the development of portable lateral flow assays (LFAs), for instance for SARS-CoV-2 testing (Chen et al. 2020; Grant et al. 2020a; Wang et al. 2020; FIND 2021a; Hristov et al. 2021). Antigen testing has gained high interest as it can be used for the detection of viruses and utilized as a control measure in communities (Grant et al. 2020a). Many tests involve detection of the highly abundant coronavirus nucleocapsid (N) and spike (S) proteins, which are considered ideal targets in antigen testing (Feng et al. 2020). For example, Hristov et al. (2021) showed the detection of coronavirus S proteins on a paper-based, sandwich-type LFA immunoassay. Mertens et al. (2020) developed an immunochromatographic assay for the rapid detection of SARS-CoV-2N proteins.

Additionally, commercial coronavirus home test kits for antigen detection are available from pharmacies or supermarkets (Coris BioConcept 2020b; Abbot 2021; FIND 2021b; Roche Diagnostics 2021; Sheridan 2020). With these tests, the possible infected sample is collected and analyzed on-site, with no need for assistance from healthcare professionals. POC-based tests enable testing with non-invasive specimens, e.g*.* saliva, which facilitates convenient sampling (Azzi et al. 2020; Han and Ivanovski 2020; To et al. 2020). Unfortunately, the performance of typical rapid tests varies considerably. The current technologies do not necessarily provide repeatable results and antigen tests are not considered as reliable as those from RT-PCR(World Health Organization (WHO) 2020). Especially, the main challenge for POC tests is the detection of small amounts of proteins. Consequently, there is a critical need for highly sensitive tests. Although specific antibodies against coronavirus antigens are available, the immobilization capacity of the former on sensor substrates can be insufficient (Yetisen et al. 2013; Shen et al. 2017).

Efforts to improve immobilization should consider the effects of physical adsorption, chemical bonding, or affinity interactions based on biomolecules (Rusmini et al. 2007). In this context, charged cellulose II nanoparticles, comprising a soft shell/hard core structure, offer the potential for controlling surface protein interactions. Specifically, we reported cationic cellulose II nanoparticles to effectively increase protein adsorption and antibody immobilization (Solin et al. 2020). Furthermore, anionic cellulose II nanoparticles offer the possibility to further control protein interactions (Beaumont et al. 2016b, 2017, 2019). Earlier reports showed that the corona of the anionic nanospheres can deform and interpenetrate into a colloidal nanogel structure (Beaumont et al. 2019). The swelling behavior of such anionic material offer promise to passivate immunoassay supports from non-specific protein adsorption given its hydrophilicity and involved electrostatic interactions. Supports that facilitate sensitive and rapid detection also need consideration. As an alternative to paper-based substrates, we have developed printable wicking materials comprising calcium carbonate particles and micro- and nanocellulose binders that are stencil-printed on flexible paper or polymer substrates, forming fluidic channels (Solin et al. 2021). These printable pastes allow tunable and complex flow channel designs. Besides, the possibility to tailor the paste composition enables modification of wicking properties, useful in regulating fluid flow for specific analyte detection.

**Fig. 1:**
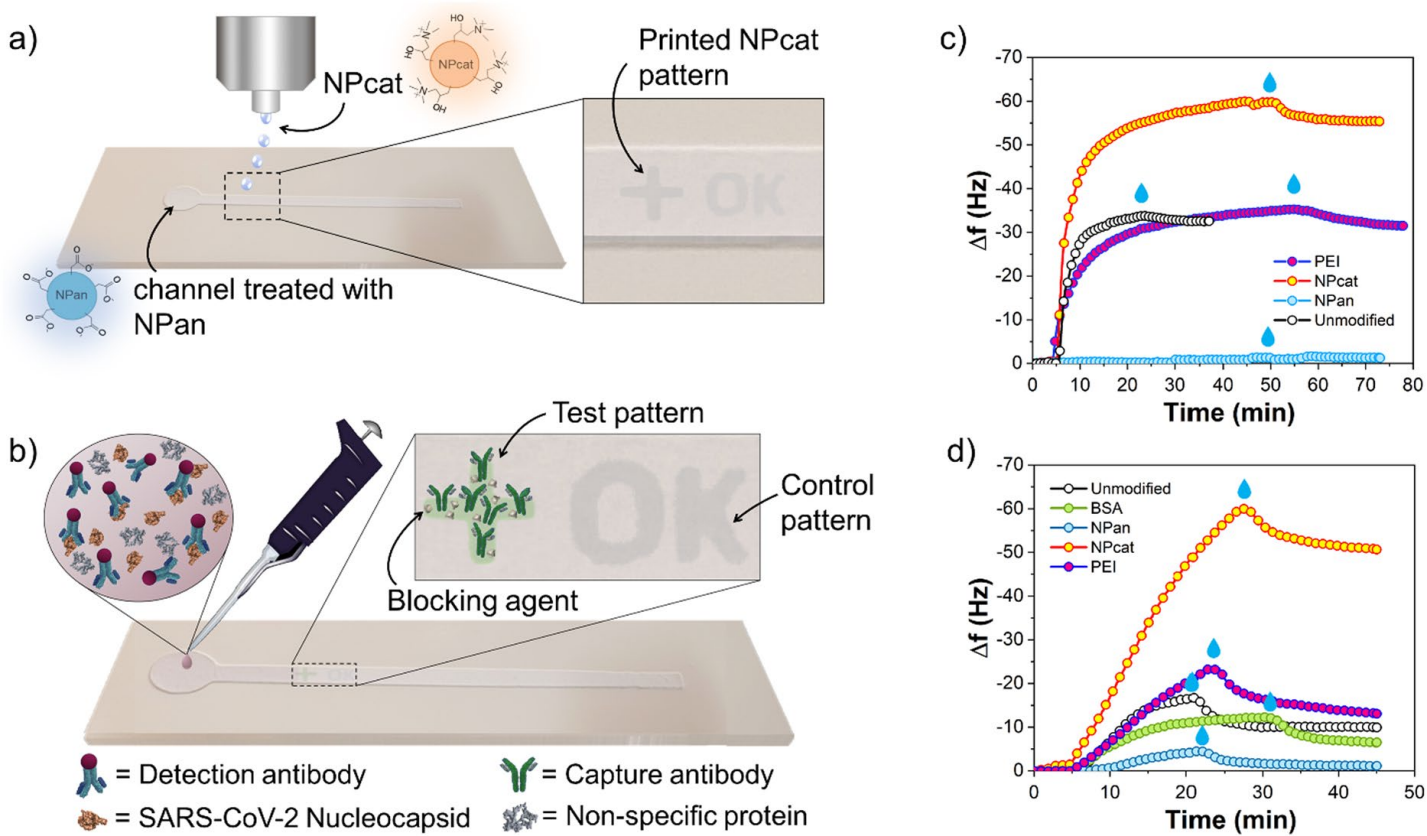
Use of cellulose II nanoparticles in a colorimetric immunoassay for detection of SARS-CoV-2 nucleocapsid on stencil printed fluidic channels: **a** NPan passivates the assay substrate from non-specific adsorption. Printed NPcat patterns form anchoring patterns on the assay sensing areas. **b** Nucleocapsid-specific capture antibodies are immobilized on the test pattern (BSA added to ensure specific detection) while the control pattern is unmodified. QCM-D sensograms demonstrate the ability of cellulose II nanospheres to control protein adsorption: **c** BSA adsorption on unmodified, PEI-, NPcat- and NPan-treated model surfaces. **d** Fibrinogen adsorption on unmodified, PEI-, NPcat- and NPan-treated model surfaces. The blue droplet symbols are added to indicate the time at which rinsing with buffer was applied

In this work, cellulose II nanoparticles are used to control protein interactions on surfaces and to enable sensitive and fast SARS-CoV-2 nucleocapsid detection. The involved interactions are investigated by using electromechanical sensing and confocal microscopy. Besides, we demonstrate the use of cellulose nanospheres in printable, flexible immunoassay systems. Cationic nanoparticles are used as an effective anchoring layer, i.e., to immobilize the sensing elements on the given support; meanwhile, negative cellulose particles act as blocking or passivating component, preventing non-specific adsorption. The assay system is prepared by stencil printing the fluid-wicking channel on flexible paper support. Moreover, inkjet-printing of the anchor layer pattern on the channel is carried out and followed by immobilization of the sensing antibodies, enabling the detection of SARS-CoV-2 nucleocapsid (see Fig. 1).

## Experimental

### Synthesis of charged cellulose II nanoparticles

Charged cellulose nanoparticles were produced following our previous reports (Beaumont et al. 2016a, 2019; Solin et al. 2020). First, 5 wt% cellulose II gel was prepared from microcrystalline cellulose (MCC) by treatment in 7 wt% NaOH and 12 wt% urea. The slurry was stored at − 20 °C overnight, thawed at room temperature, diluted to 1 wt%, and equilibrated for 1 h. Finally, the sample was washed by dialysis. *Cationic cellulose II nanoparticles (NPcat)*: The cellulose II gel was concentrated (10,000 rcf, 10 min) to a solids content of ca. 9 wt%. The suspension (32 g) was mixed with a 50 wt% aqueous solution of NaOH (0.38 g, 4.8 mmol). After 30 min, glycidyltrimethylammonium chloride (5.5 mL, 6.2 g) was added and the viscous gel was transferred into a water bath at 55 °C. After 20 h, the suspension was washed with MilliQ water following three washing and centrifugation cycles (5 min at 10,000 rcf). The suspension was further purified by dialysis for 4 days. Finally, the suspension was homogenized (2 passes at 2000 bar, Microfluidics M110P, Microfluidics Corp.) to yield individualized spherical nanoparticles. *Anionic cellulose II nanoparticles (NPan)*: 100 mL of cellulose II gel (4.0 wt%) was concentrated by centrifugation at 10 000 rcf for 10 min. Then, the concentrated slurry was diluted with 100 mL of 2-PrOH, filtrated, and washed with 2-PrOH (3 $\times \hspace{0.17em}$100 mL). Next, 1.3 M NaOH was added to the suspension (4 wt%). After equilibration for 30 min, 2.9 g of sodium chloroacetate was added and stirred at 55 °C for 4.5 h. The product was filtered and washed intensively with MilliQ water by a set of washing and centrifugation (5 min at 4000 rcf) until gaining neutral pH. Finally, the centrifugation time was increased to 30 min. After washing, the suspension was diluted to a solids content of 1.4 wt% and homogenized at 800 bar using 4 cycles. This suspension was centrifugated for 5 min at 4000 rcf to remove small amounts of residual, coarse particles. Further nanoparticle characterization and a description of associated methods are provided in the Supporting Information document (SI). In addition, further characterization of cellulose II nanoparticles has been performed in earlier publications (Beaumont et al. 2016a, b, 2017, 2019; Solin et al. 2020).

### Stencil printing of fluidic channels on paper

To prepare porous substrates for immunoassays, fluidic channels were stencil printed on PowerCoat® paper supports according to our previously reported method (Solin et al. 2021). Briefly, a Ca-CH paste was prepared by mixing CaCO_3_, CNF, and HefCel in DI water (dry weight ratio 95:2.5:2.5), and propylene glycol (5 wt% of the wet paste) was also added. Finally, fluidic channels were stencil printed on the substrate, as illustrated in Figure S1. A detailed description of the channel preparation is found in the SI document.

### Preparation of model films for quartz crystal microgravimetry

To study protein interactions on the printed fluidic channels, thin model films were prepared from the diluted Ca–CH paste (37–22.8 wt% solids content). Notably, before Ca–CH paste preparation, CaCO_3_ was ball-milled for 40 min to decrease the particle size and subsequently filtered. The diluted sample was drop-cast onto UV-ozonized QCM-D crystals (QSX 301 Au, Biolin Scientific) with a thin anchor layer of polyethyleneimine (PEI) and washed with MilliQ to remove any loosely bound material.

### Quartz crystal microbalance with dissipation monitoring (QCM-D)

QCM-D (E4 instrument, Q-Sense AB) was employed to monitor protein adsorption on the model surfaces at pH 7.4. Changes in the sensor oscillation frequency were measured at a fundamental resonance frequency of 5 MHz. All measurements were performed at 23 °C, under a constant flow of 100 μL/min. Each sample was measured at least twice. Before measurement, the model films were stabilized in a phosphate buffer (pH 7.4, 10 mM) until a stable baseline was obtained. Additional information related to data analysis and mass calculations can be found in the SI document.

The effect of NPcat and NPan on non-specific protein adsorption was studied and their performance was compared with typical anchoring and blocking materials. First, adsorption of 0.01 mg/mL BSA was monitored on unmodified, PEI-, NPcat- and NPan-treated model surfaces. The cationic materials were used as anchor layers to increase adsorption on the surfaces, whereas anionic nanoparticles were used as blocking material. The material treatments were done by injecting 0.5 wt% aqueous solutions of each material onto the model films in situ in the QCM unit before the adsorption of the proteins. Similarly, adsorption of 0.01 mg/mL fibrinogen was monitored on unmodified, PEI-, NPcat-, bovine serum albumin (BSA)- and NPan-treated model surfaces. Finally, non-specific adsorption of SARS-CoV-2 nucleocapsid (6 ng/mL) was measured on BSA-blocked model surfaces. Notably, buffer washing was done after each adsorption step.

In addition, specific interactions of SARS-CoV-2 nucleocapsid (N protein) were investigated using unmodified, PEI- and NPcat-treated model surfaces. The cationic materials were used as immobilization agents for the capture antibodies (20 µg/mL in buffer, pH 7.4). 10 µg/mL BSA solution was used to block the remaining non-specific binding sites. Then, the adsorption of N protein (6 ng/mL) and detecting antibody (20 µg/mL) was monitored. We also studied the adsorption behavior of human immunoglobulin G (hIgG) to its secondary antibody, anti-hIgG. First, 20 µg/mL anti-hIgG was adsorbed on the model surfaces. After BSA blocking (10 µg/mL), adsorption of 10 µg/mL hIgG was investigated. Notably, washing with buffer was done between each adsorption step.

### Protein adsorption and confocal laser scanning microscopy (CLSM)

To further demonstrate the immobilization capability of NPcat and the blocking effect of NPan, the non-specific adsorption behavior of hIgG-FITC (see SI document) was studied on filter paper and printed fluidic channels. 3 µL of 0.5 wt% PEI, NPcat, or NPan were adsorbed on the substrates. Then, substrates were washed with MilliQ; filter papers were fully immersed in water and the printed channels were washed by pipetting water on the channel. Next, 10 µL of 0.1 mg/mL hIgG-FITC (in buffer, pH 7.4) was adsorbed on the samples, followed by washing. Finally, the hIgG-FITC exposed samples were imaged with CLSM to detect the adsorbed antibodies. Images were taken with a laser scanning spectral confocal microscope (Leica TCS SP2, Leica microsystems CMS GmbH) by using a 488 nm excitation wavelength and 500–540 nm detection wavelength range. Images were acquired using a 750 V laser power and under constant imaging conditions. Two replicates of each sample were imaged. The intensity of fluorescence of each confocal image was determined using Adobe Photoshop 2021.

### Inkjet-printing of patterns with cationic materials

An inkjet printer (Dimatix Materials Printer, DMP-2831, Fujifilm) was used to print NPcat and PEI patterns on top of filter paper, nanopaper, and printed channels. 0.5 wt% NPcat solution was filled in DMC-11610 cartridges and printed using a drop spacing of 20–40 μm at 2 kHz frequency, 35 V jetting voltage, and 3-inch H_2_O meniscus vacuum. Alternatively, 0.5 wt% PEI solution was printed on the substrates with a drop spacing of 20–40 μm at 3 kHz frequency, 24 V jetting voltage, and 3-inch H_2_O meniscus vacuum. Various designs and pattern sizes were tested and 1–10 layers of material were printed. The adsorption capabilities of printed NPcat and PEI patterns were studied with fluorescein-based dye and fluorescent-labeled proteins (experimental details are provided as Supporting Information).

### Coronavirus antigen detection with patterned sensors

*Preparation of the assay*: Fluidic channels were treated with NPan. Then, NPcat patterns were inkjet-printed (40 μm drop spacing, 2 kHz frequency, 35 V jetting voltage, 3-inch H_2_O meniscus vacuum) on the test and control areas. Two NPcat layers were printed on the test area forming a “+” pattern (2.2 $\times \hspace{0.17em}$2.2 mm^2^) and three layers were printed on the control area to form an “OK” pattern (3.5 $\times$ 2 mm^2^), Figure S2. 3 µL of capture antibody (0.1 mg/mL) was drop-cast on top of the test pattern. The control pattern (“OK”) was either left untouched or 3 µL of anti-mouse IgG (0.5 mg/mL) was dropped on top of the printed pattern. Washing was carried out with a constant volume of buffer (pH 7.4, 10 mM) by using a pipette to remove loosely bound molecules. Next, BSA (0.5 wt%) was used to block the remaining uncovered NPcat areas on the test zone, and also on the control zone if antibodies were applied. Finally, washing was done with a constant volume of buffer and the assays were dried before testing. *Coronavirus antigen sensing:* Detection of the SARS-CoV-2 nucleocapsid was performed with the dried assays and each experiment was repeated at least three times. The analyses were first performed by testing antigen-positive (0.4–8 ng/mL N protein, 2 wt% casein hydrolysate, 1 wt% fibrinogen, and AuNP-labeled detection antibodies (OD = 0.5)) and antigen-negative (2 wt% casein hydrolysate, 1 wt% fibrinogen, detection antibodies (OD = 0.5)) samples in the buffer. Additionally, saliva samples were tested and compared with commercial testing units, which were purchased from a local pharmacy. An antigen-negative sample was prepared by mixing saliva and buffer with AuNP-labeled detection antibodies (1:1 ratio). The positive sample was prepared by mixing saliva with N protein (1 µg/mL), buffer, and AuNP-labeled detection antibodies (1:1 ratio). All samples were analyzed by drop-casting 40 µL of solutions on the circular sample area of the prepared assays. Analysis of the results was done after 2–15 min. A positive result was indicated with color development in both, the control and test areas. A negative result was indicated with color development in the control area.

## Results and discussion

### Cellulose II nanoparticles

Following the previous publications(Beaumont et al. 2016a, b, 2019; Solin et al. 2020), the introduction of repulsive charges to amorphous regions of cellulose II hydrogels and the combination of mechanical treatment produced charged soft cellulose II nanospheres. Anionic cellulose II nanoparticles (NPan) were obtained by carboxymethylation, whereas cationic cellulose II nanoparticles (NPcat) were produced following a reaction with glycidyltrimethylammonium chloride. The FTIR spectra of the nanoparticles (Figure S3), colloidal properties (Table S1), and AFM images (Figure S4) are supplied as SI. The unique feature of the hard core/soft shell cellulose nanospheres involves self‐assembly into densely packed colloidal nanogel layers, which are applied to control protein interactions on cellulose thin films and paper(Beaumont et al. 2019; Solin et al. 2020).

### Protein interactions

QCM-D was used to monitor protein adsorption and interactions between the coronavirus antigen and sensing elements, which would later be employed in antigen-sensing immunoassay. The measurement was done on thin films used as models of the printed fluidic channels (SEM images in Figure S5). Noteworthy, the prepared model films were not as porous as the macroscale fluidic channels (Figure S6); therefore, the effect of the three-dimensional structure on adsorption could not be observed with this method.

Non-specific binding of BSA was studied with unmodified, PEI-, NPcat- and NPan-treated model surfaces to demonstrate the ability of the nanoparticles to control protein interactions. The frequency changes of the oscillating QCM sensors, after BSA injection, can be seen in Fig. 1c. The calculated adsorbed protein masses are included in Table 1 and the measured shifts in energy dissipation are listed in Table S2. The adsorbed protein mass was estimated with the Voigt model to correlate the viscoelastic changes with the protein mass (see experimental details in the Supporting Information). For comparison, the Sauerbrey(Sauerbrey 1959) approximation was also used. With the Voigt model, 637 ng/cm^2^ BSA adsorption was obtained on the unmodified surface. Besides, NPcat significantly increased the adsorption of BSA, by 111% (1345 ng/cm^2^). By contrast, NPan reduced the interactions, by 98% (14 ng/cm^2^). BSA adsorption on the PEI-treated surface was 19% lower (515 ng/cm^2^) compared with the unmodified surface.

**Table 1:** Calculated BSA and fibrinogen adsorbed mass on the given model surface

Surface	Voigt (ng/cm^2^)	Sauerbrey (ng/cm^2^)
*BSA*		
Unmodified	637	576
PEI	515	571
NPcat	1345	981
NPan	14	23.8
*Fibrinogen*		
Unmodified	188	176
PEI	205	232
NPcat	858	896
NPan	18	19.9
BSA	137	113

Protein interactions were studied with another non-specific protein, fibrinogen. Fibrinogen adsorption isotherms on the unmodified, BSA-, NPan-, PEI-, NPcat-treated surfaces are included in Fig. 1d, and the calculated adsorbed masses are listed in Table 1. The measured dissipation changes are shown in Table S2. The adsorption of fibrinogen on the unmodified surface was 188 ng/cm^2^, while PEI- and NPcat-treated surfaces adsorbed 205 (+ 9%) and 858 ng/cm^2^ (+ 356%), respectively. The blocking efficiency of NPan was compared with BSA, a commonly used blocking agent. NPan blocked fibrinogen adsorption more effectively, by 90% (18 ng/cm^2^), compared to the unmodified surface or the BSA-blocked surface (137 ng/cm^2^, − 27%).

**Fig. 2:**
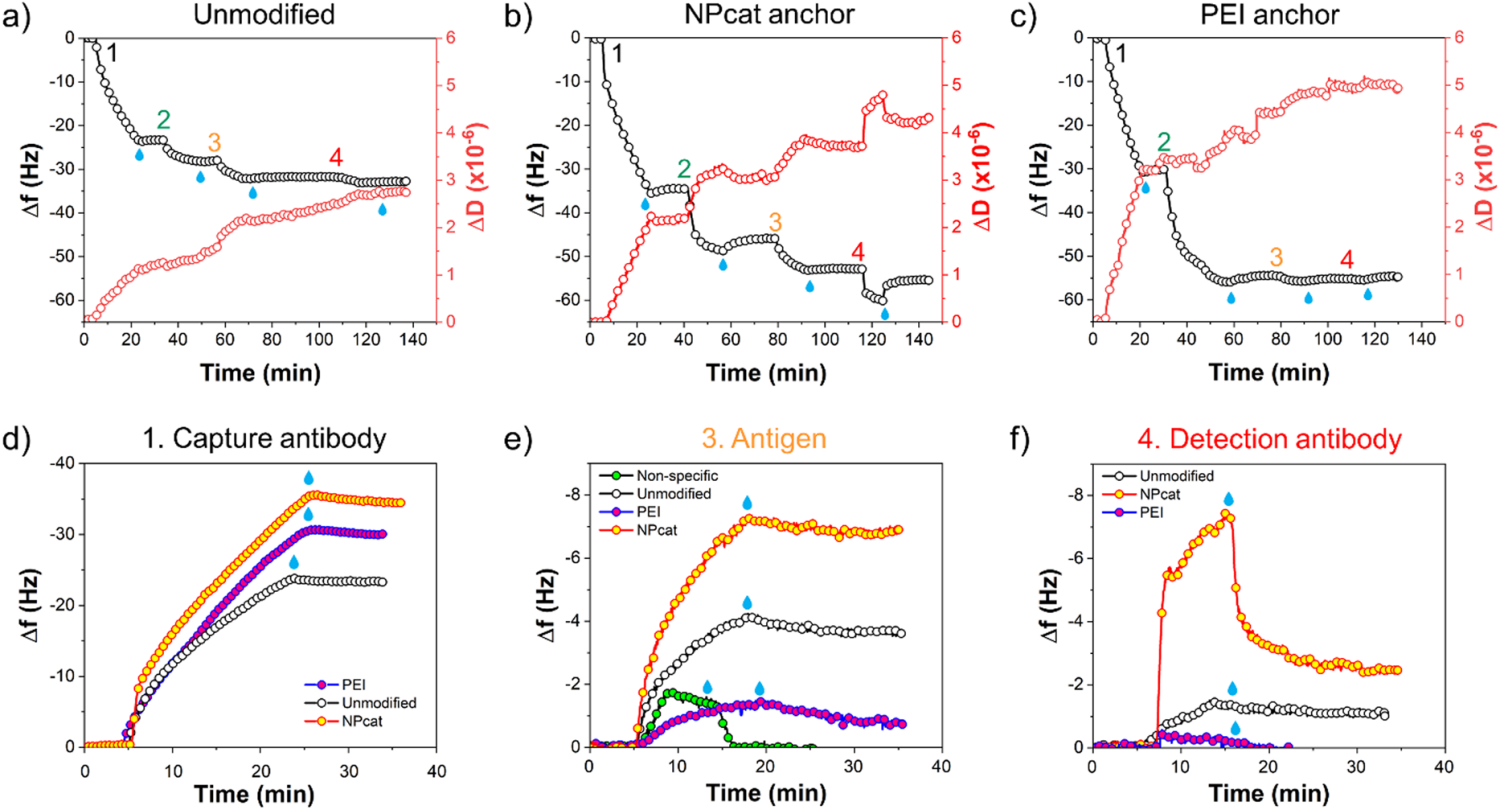
QCM-D sensograms upon adsorption of (1) capture antibody, (2) BSA, (3) SARS-CoV-2 nucleocapsid, and (4) detection antibody on **a** unmodified, **b** NPcat-treated and **c** PEI-treated model surfaces. The effect of surface treatments on the detection can be seen by comparing the adsorption steps: **d** adsorption of capture antibody on unmodified, PEI-, and NPcat-modified surface, **e** adsorption of antigen (N protein) after BSA-blocking on each surface, adsorption curve for non-specific antigen binding to BSA-blocked model surface is given as comparison, **f** adsorption of detection antibody on each surface. The blue droplets indicate rinsing with buffer

The QCM-D technique was also employed to monitor coronavirus antigen detection. The studied interactions between antibodies and N protein were later utilized in the development of coronavirus antigen test since they occur on the test area of the assay. Figure 2a–c shows the measured frequency and dissipation changes upon antibody and antigen interactions with the unmodified, NPcat- and PEI-treated surfaces (AFM images in Figure S7). First, (1) capture antibodies were adsorbed on each surface. Then, (2) BSA was used for blocking. (3) Specific adsorption of the antigen (SARS-CoV-2 N protein) and (4) antibody detection were assessed. To show the difference in binding of each surface, the cut QCM-D data of antibody and antigen adsorptions are presented in Fig. 2d–f. Both cationic materials increased the binding of the capture antibody. The calculated adsorbed mass is listed in Table 2. Compared to the adsorbed mass on the unmodified surface (474 ng/cm^2^), we measured a 64% higher adsorption of the capture antibody on PEI-treated surface (777 ng/cm^2^) and 83% higher on the NPcat-treated surface (867 ng/cm^2^).

**Table 2:** Adsorbed mass of the coronavirus antibodies and antigen on each surface

Surface	Adsorbed protein	Δ*m*_Voigt_ (ng/cm^2^)	Δ*m*_Sauerbrey_ (ng/cm^2^)
Unmodified	Capture antibody	474	412
	N protein	115	63
	Detection antibody	27	18
NPcat-treated	Capture antibody	867	609
	N protein	194	119
	Detection antibody	88	45
PEI-treated	Capture antibody	777	529
	N protein	28	14
	Detection antibody	–	–

The binding of antigen was analyzed on each surface after BSA treatment. The antigen mass obtained for unmodified, PEI- and NPcat-treated surfaces, corresponds to 115, 28, and 194 ng/cm^2^, respectively. Thus, the highest adsorption of N protein was obtained with NPcat-treated supports (Fig. 2e). Most likely, improved immobilization of the capture antibody led to higher antigen binding. However, PEI-treated surface showed limited adsorption of the antigen, despite the increased antibody binding. Possibly, the PEI surface adsorbed the capture antibodies in a non-active conformation, reducing the binding of the antigen. In addition, Fig. 2e shows only minor non-specific adsorption of the antigen on the BSA-blocked model surface. Furthermore, the NPcat surface facilitated better adsorption of the detection antibody (88 ng/cm^2^) compared to the unmodified surface (27 ng/cm^2^) (Fig. 2f). The PEI-treated surface did not adsorb significant amounts of the detection antibody. Moreover, we studied the effect of cationic treatments on hIgG interactions with its secondary antibody, anti-hIgG (Figure S8). These kinds of interactions typically occur on the control line of an immunoassay. Similarly, increased immobilization capability was obtained with the NPcat treatment compared to the unmodified and PEI-treated surfaces.

### Protein adsorption and confocal microscopy

**Fig. 3:**
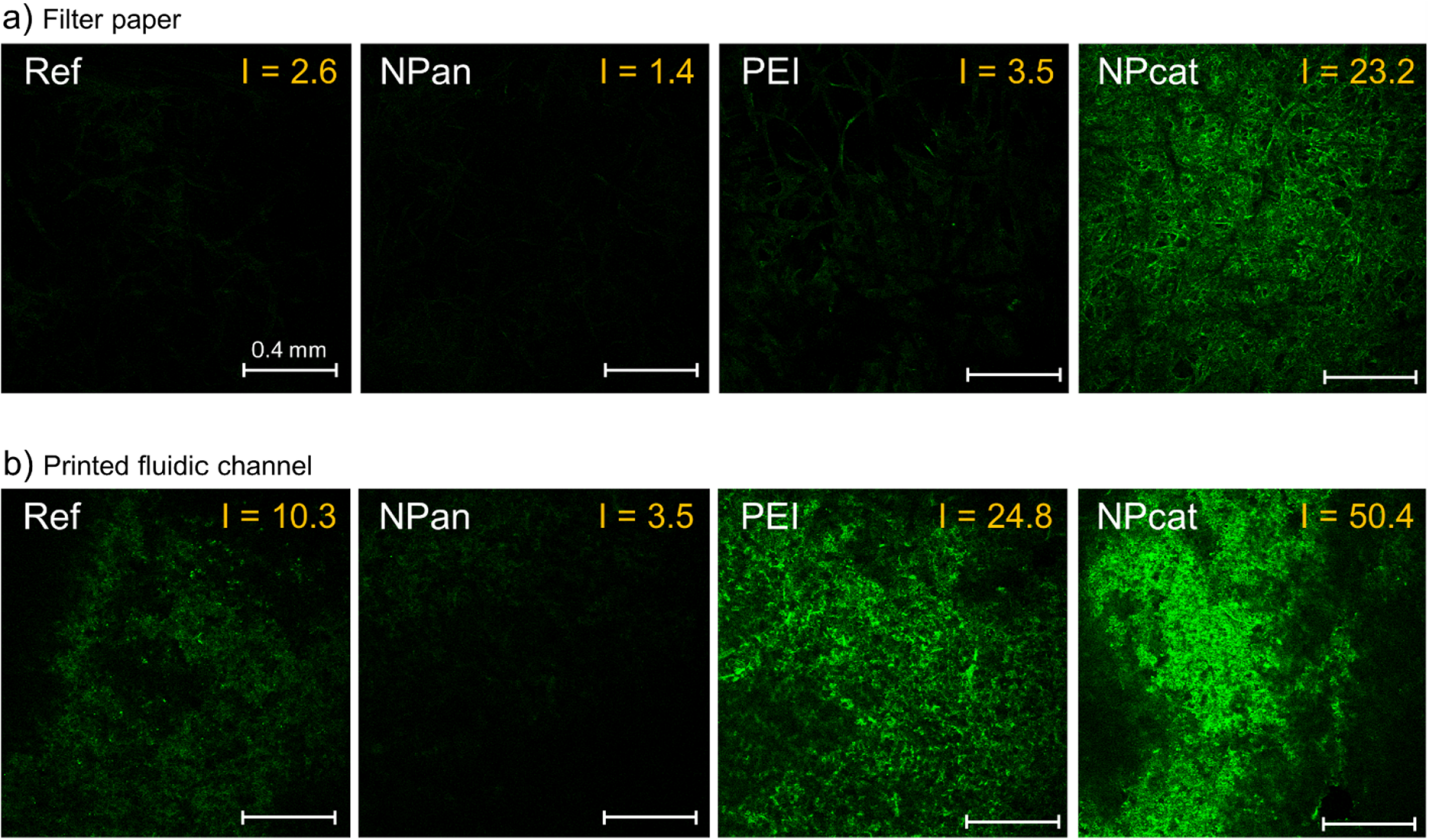
Confocal images of **a** filter paper and **b** printed fluidic channels exposed to fluorescent-labeled proteins (hIgG-FITC) and the effect of surface treatments on protein adsorption. The fluorescence intensity, *I*, is indicated in each image. Scale bar in each image 0.4 mm

To translate the QCM-D results to paper-based systems, the effect of surface treatment on antibody adsorption was studied by CLSM employing filter papers and printed fluidic channels. Non-specific adsorption of human IgG with a fluorescent tag (hIgG-FITC) can be seen on unmodified (Ref), NPan-, PEI, and NPcat-treated substrates in Fig. 3. The test results on filter paper (Fig. 3a) showed only minor antibody adsorption on the unmodified surface (*I* = 2.6), and even smaller quantities were detected after NPan treatment (*I* = 1.4), demonstrating its blocking efficiency. Oppositely, both cationic treatments increased adsorption, and NPcat showed a significantly higher fluorescence intensity (*I* = 23.2) compared to PEI (*I* = 3.5). The effect of the treatments on protein adsorption followed the same trend on the printed channels (Fig. 3b) with generally higher fluorescence intensity (unmodified channel *I* = 10.3). This difference is most likely related to the washing procedures of the substrates. We also compared the blocking effect of NPan with BSA (Figure S9). BSA had only a minor effect on non-specific adsorption and quite similar intensity of fluorescence as that for the unmodified substrate (*I* = 9.2 on printed channel and *I* = 2.2 on filter paper).

### Inkjet-printing of cationic patterns

We studied the printing capability of PEI and NPcat on filter papers, printed fluidic channels, and nanopapers. The successful deposition of the cationic inks was visualized by the adsorption of a negatively charged dye on the substrates with the printed patterns (Figure S10). In comparison to NPcat, PEI prints were blurry and had lower resolution. Moreover, by using confocal imaging, we determined high antibody adsorption on printed NPcat and PEI patterns. Figure S11 shows images of the unmodified, PEI- and NPcat-patterned nanopapers exposed to fluorescent-labeled antibodies. Both non-specific (Figure S11a, c, e) and specific (Figure S11b, d, f) interactions are shown. In addition, the confocal images of the NPcat and PEI patterns without the fluorescent-tagged antibodies had only low background fluorescence, Figure S12.

### Detection of coronavirus protein

**Fig. 4:**
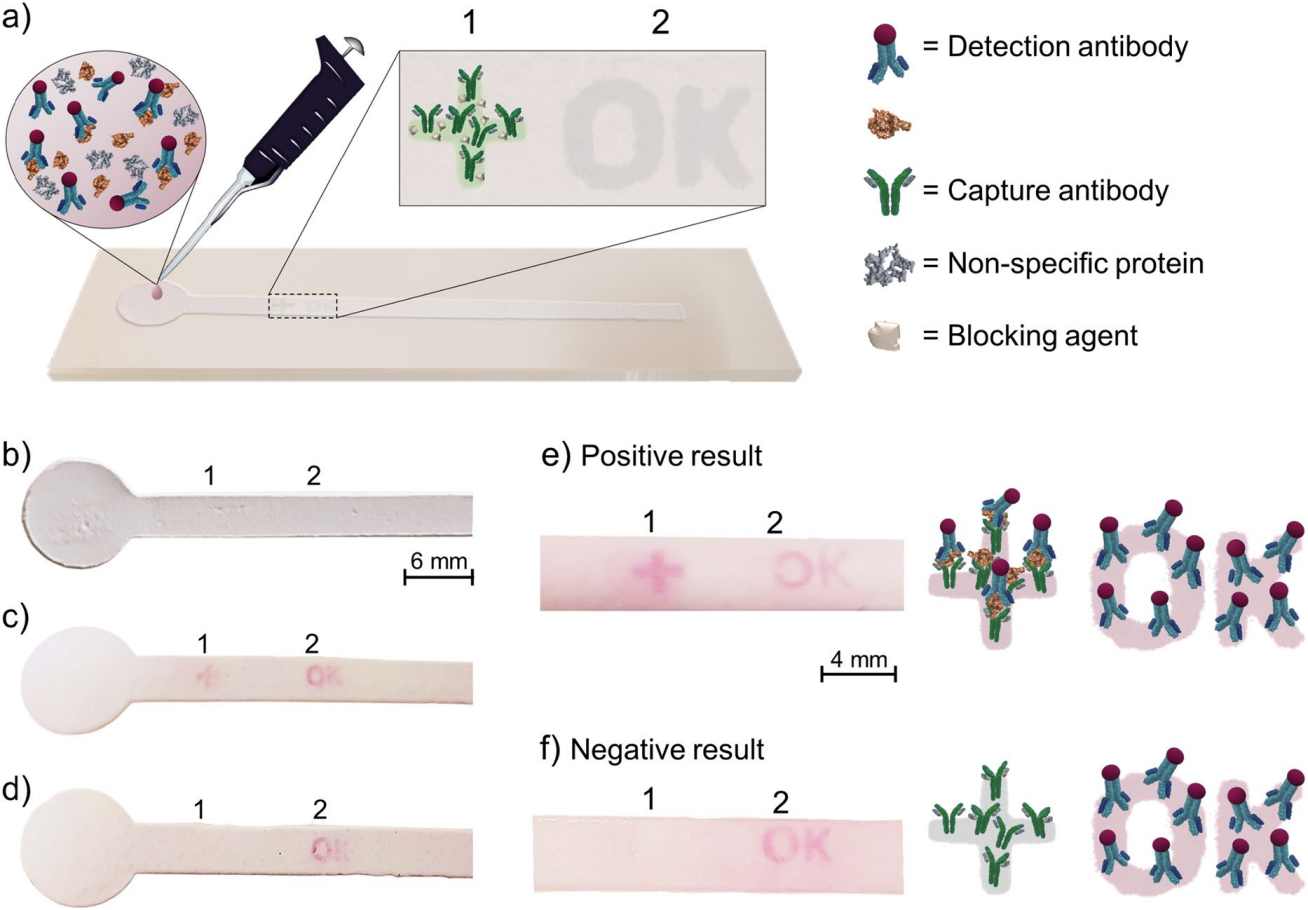
Performance of the introduced, rapid SARS-CoV-2 antigen test: **a** Schematic illustration of the prepared assay: Printed NPcat patterns formed the test (“+”) and control (“OK”) patterns of the sensor. The test area (1) was treated with capture antibodies and blocker while the control area (2) was left untouched. The assays were tested with antigen-positive, and antigen-negative samples. Images of cropped immunoassays showing colorimetric responses before and after testing: **b** untested assay with marked positions of the test (1) and control (2) areas (patterns invisible), tested and washed assays exposed to **c** antigen-positive sample (8 ng/mL N protein) and **d** antigen-negative sample. Schematic illustrations of the protein interactions on the sensing area and corresponding color development in unwashed assays: **e** antigen-positive sample and **f** antigen-negative sample. The optical density of all samples was 0.5

Coronavirus antigen tests were prepared on the printed channels by employing a sandwich immunoassay system after few alterations, Figs. 1 and 4. Usually, in this type of assay, the analyte is absorbed and transported along a paper strip with antibodies with a detectable label. In this work, printed fluidic channels deposited on PowerCoat® paper (Figure S6, Figure S13) were used as a substrate. The fluid-wicking properties of these channels have been analyzed in our previous work, where we reported that the channels wicked 4 cm of water in approx. 130 s (Solin et al. 2021). Here, the substrate was passivated from non-specific protein adsorption with NPan. Then, NPcat was used to print anchor patterns on the assay sensing area to immobilize capture antibodies. As shown in Fig. 4a, the capture antibodies were placed on the test zone on top of the test pattern (“+”), but the control pattern (“OK”) was left untouched. Noteworthy, the test pattern was blocked carefully with BSA to ensure specific detection of the N protein and to prevent false-positive results. In this test system, the detection antibodies were mixed directly in the sample solution instead of a typical, separate conjugate pad layout (Fig. 4a). Furthermore, since proteins have a high affinity with NPcat, the detection of the control pattern was enabled by the non-specific binding of the detection antibody to the NPcat pattern. However, we also tested a sensor system with deposited secondary antibodies on the control pattern. Both positive and negative results could similarly be obtained with this approach. The test results of this system and illustrations of the possible interactions are included in Figure S14.

Figure 4b–f show cropped images of the assays and the test results (images with the background in Supporting Information). There are no visible patterns on the untested assay. However, after testing antigen-positive (Fig. 4c, e) and antigen-negative (Fig. 4d, f) samples, appropriate color patterns developed. The positive sample with 8 ng/m N protein caused a reaction on both test and control patterns while the negative sample caused color only on the control pattern. The formation of the color was very fast, and a readable result could be obtained within 2 min. Still, to ensure the formation of a negative result, a 10-min analysis time was applied. Furthermore, the background adsorption on the test substrate was quite low after washing the samples (Fig. 4c, d). A pale pink color could be seen before washing (Fig. 4e, f) despite the NPan blocking, which is reasoned by the used test layout, which requires a separate washing step to remove loosely bound proteins.

The significance of the nanoparticle treatments on sensor performance is highlighted in Figure S16, where cropped images of the assays with and without nanoparticle treatments are presented. If the substrate was not blocked with NPan, significant non-specific adsorption took place even after washing (Figure S16a). A high amount of detection antibodies adsorbed especially on the edges of the channels, and strong color developed on the NPcat patterns. Background adsorption and non-specific binding on the test pattern were prevented by NPan and BSA blocking, and deposition of the capture antibody (Figure S16b). The use of NPcat as an anchor layer is required, as shown in Figure S16c. No detectable signal could be observed without immobilization of the sensing antibodies with NPcat.

Moreover, an estimation of the detection limit of the developed sensor was obtained optically. Figure S16d shows a gradual fading of the test pattern with decreasing N protein concentration. The lowest detectable concentration was 0.4 ng/mL, which produced a faded “+” pattern within 5 min. To put this result in context, we note detection limits of commercial devices of 0.25 (Coris BioConcept 2020a), 0.65 (Grant et al. 2020b), and 100 (Lomina 2021) ng/mL obtained after 10–30 min (Coris BioConcept 2020a; AccessBio 2021; Lomina 2021; Barlev-Gross et al. 2021). We note that proper determination of the limit of detection, LOD, requires tests under field conditions and accurate signal quantification, which were not attempted in this proof-of-concept study. The assay performance was also analyzed with saliva samples and the results were compared with commercial diagnostic devices, “*Device A*” and “*Device B*” (Figure S17). Our developed assay performed quite effectively with human saliva. Three parallel antigen-positive samples showed three positive results and three antigen-negative samples produced three negative results. The test results were obtained in approx. 3 min. When testing the performance of commercial tests, negative results were obtained but, in some samples, it was difficult to produce distinctive positive results. For example, *Device A* (Figure S17b) showed only one faded positive result in 10 min and two false-negative results (three parallel samples, reported test time 10–20 min). On the other hand, *Device B* (Figure S17c) performed well and produced two clear positive results in approx. 2 min and one faded positive result in 9 min (three parallel samples, reported test time 15 min). Finally, we acknowledge that further analysis and optimization are needed to conclude any clinical relevance. Herein, we assessed the use of cellulose nanoparticles, in a cellulose-based substrate, to demonstrate a promising platform. Approved protocols, testing of viral samples and actual conditions of use should be considered to inform any field application.

## Conclusions

We demonstrated the use of cellulose II nanoparticles to control protein interactions on surfaces. QCM-D measurements and fluorescent imaging confirmed that the nanoparticles enable a better performance compared to typical materials. Effective passivation towards non-specific proteins was obtained with NPan, while improved protein adsorption, as well as effective immobilization of sensing antibodies, was achieved with NPcat. Additionally, NPcat was conveniently inkjet-printed on various substrates, forming patterns with high adsorption capability. Utilizing the cellulose nanospheres on patterned immunoassays enabled rapid and sensitive SARS-CoV-2 nucleocapsid detection. The developed prototype sensor showed excellent performance in testing with saliva samples, developing a signal in a short time, three minutes. Considering that this sensor is an early demonstration and prototype, the cellulose II nanospheres are shown for their promise in enabling rapid and sensitive immunoassays.

## Supplementary Information

Below is the link to the electronic supplementary material.

Supplementary file1 (DOCX 17022 kb)

## Data Availability

The authors confirm that the data supporting the findings of this study are available within the article.
