# Supplementary material for: Immobilized cellulose nanospheres enable rapid antigen detection in lateral flow immunoassays
Source: Cellulose (Lond). 2023 Jan 5;30(4):2353–65. doi: 10.1007/s10570-022-05038-y (PMC9813465; doi:10.1007/s10570-022-05038-y)
Supplement: Supplementary file 1 — Supplementary file1 (DOCX 17022 kb) [file 10570_2022_5038_MOESM1_ESM.docx]

**SUPPORTING INFORMATION**

**Immobilized cellulose nanospheres enable rapid antigen detection in lateral flow immunoassays**

Katariina Solin, Marco Beaumont, Maryam Borghei, Hannes Orelma, Pascal Mertens, and Orlando J. Rojas*

K. Solin, M. Borghei, O.J. Rojas

Department of Bioproducts and Biosystems, School of Chemical Engineering, Aalto University, Vuorimiehentie 1, FI-00076, Espoo, Finland

K. Solin, H. Orelma

VTT Technical Research Centre of Finland Ltd., Tietotie 4E, FI-02044 Espoo, Finland

M. Beaumont

Department of Chemistry, Institute of Chemistry for Renewable Resources, University of Natural Resources and Life Sciences Vienna (BOKU), Konrad-Lorenz-Straße 24, A-3430 Tulln, Austria

P. Mertens

Coris BioConcept, Rue Jean Sonet 4A, B-5032 Gembloux, Belgium

Prof. O. J. Rojas

The Bioproducts Institute, Departments of Chemical and Biological Engineering, Chemistry and Wood Science, University of British Columbia, 2360 East Mall, Vancouver, BC, V6T 1Z4 Canada.

E-mail: orlando.rojas@ubc.ca

**SI Table of Contents**

Materials and additional methods S3

Figure S1 S5

Figure S2 S7

Figure S3 S7

Table S1 S8

Figure S4 S8

Figure S5 S9

Figure S6 S9

Figure S7 S10

Table S2 S11

Figure S8 S12

Figure S9 S13

Figure S10 S14

Figure S11 S15

Figure S12 S16

Figure S13 S16

Figure S14 S17

Figure S15 S18

Figure S16 S19

Figure S17 S20

References S21

**Materials and additional methods**

**Materials.** Cellulose nanofibrils (CNF) were prepared from bleached Kraft birch pulp fibers by using a microfluidizer (M110P fluidizer, Microfluidics Corp.) equipped with 200-μm and 100-μm chambers operated at 2000 bar using six passes. High-consistency enzymatic fibrillated cellulose (HefCel) and nanopaper (CNF films) were provided by VTT Technical Research Centre of Finland(Tammelin et al. 2013; Pere et al. 2020). Microcrystalline cellulose (Avicel) was purchased from Merck. PowerCoat® HD was provided by Guarro Casas(2020). Antibodies directed towards SARS-CoV-2 nucleocapsid protein were previously optimized and characterized(Bussmann et al. 2006; Mertens et al. 2020). A humanized monoclonal antibody (MAT-8523, antibody A) was used as a capture antibody. Mouse monoclonal antibody (MAT-8524, antibody J) was used as a detection antibody after passive adsorption at 27 µg/mL on gold nanoparticles NP-AU4-529 (NanoQ, Belgium). Gold nanoparticles concentration was measured by optical density at 529 nm. Notably, the concentration for 1 ml of conjugate at 1 OD529 nm was 6.76 µL/mL of the detection antibody. At this concentration of beads (1 OD 529 nm), the estimate is that there are 7.15x10^10^ beads per ml. Beads are 40 nm in diameter. The surface concentration of antibody on the beads was approx. 19 ng/mm^2^. Gene encoding SARS-CoV-2 recombinant nucleoprotein was cloned into pET-21a(+) vector and protein was overexpressed in E. coli BL21(DE3). After centrifugation, the bacterial pellet was resuspended in PBS and sonicated, and soluble extract containing the protein was collected by centrifugation. This crude soluble fraction was used as SARS-CoV-2 antigenic positive control. Also, SARS-COV-2 Nucleocapsid (aa1-419) His Tag Recombinant protein and Spectrum™ Spectra/Por™ 6 Pre-wetted standard RC dialysis tubing (MWCO 1-50 kD) were purchased from ThermoFisher Scientific. All other chemicals were purchased from Sigma‐Aldrich: polyethyleneimine (PEI) (Mw 600 000–1 000 000 g/mol), glycidyltrimethylammonium chloride (≥90 %), sodium chloroacetate (≥98 %), human immunoglobulin G (hIgG) (≥95 %), anti-human immunoglobulin G (anti‐hIgG) γ‐chain specific antibody produced in rabbit, anti-mouse IgG (Fab specific) antibody produced in goat, fibrinogen from human plasma (50-70 % protein), casein hydrolysate, and bovine serum albumin (BSA) (≥98 %). Water purified with a Millipore Synergy UV unit (MilliQ) was used throughout the experiments.

**Nanoparticle characterization.** *Zeta-potential and particle size:* Zetasizer (Zeta sizer Nano ZS 90, Malvern) was used to measure the zeta-potential and particle size using 0.05 wt% NPan and NPcat suspensions in 2.5 mM NaCl. Six replicates were measured with each sample. *Atomic force microscopy (AFM)*: AFM (MultiMode 8 Scanning Probe Microscope, Bruker AXS Inc.) was used to analyze the surface topography of the NPcat and NPan particles deposited on SiO_2_ wafers. Surface areas of 5 x 5 μm^2^, 3 × 3 μm^2^ and 1 × 1 μm^2^ were investigated in air by using AFM tapping mode with silicon cantilevers (NSC15/AIBS, MicroMasch). Three different areas on each sample were imaged and flattening was used for image processing. *Fourier-transform infrared spectroscopy* (*FTIR):* To show successful modifications, infrared spectra of the freeze-dried nanoparticles was measured with the FTIR (Spectrum Two FT-IR Spectrometer, PerkinElmer). In addition, further characterization of cellulose II nanoparticles has been performed in earlier publications(Beaumont et al. 2016a, b, 2017, 2019; Solin et al. 2020).

**Stencil printing of fluidic channels on paper.** Stencil-printable paste was prepared by mixing CaCO_3_, CNF, and HefCel. First, CaCO_3_ was dispersed in DI water. Then, CNF and HefCel were mixed with the CaCO_3_ paste until a homogeneous dispersion was obtained. The prepared paste is hereafter referred to as Ca-CH, denoting a composition that included CaCO_3_, CNF, and HefCel at a dry weight ratio of 95:2.5:2.5. This paste had 10 g of total solids in the dry state, whereas the solids content of the wet paste was 37.0 wt%. Besides, to improve adhesion to the hydrophobic PowerCoat^®^ paper, propylene glycol (5 wt% of the wet paste) was added to the paste formulation. Finally, fluidic channels were printed on PowerCoat^®^ paper using a stencil with desired pattern dimensions. A squeegee (RKS HT3 Soft) was used to transfer the paste on paper through a plastic stencil (350 µm thickness) following a circular pattern (r = 5 mm) used as a sample deposition area. A rectangular section (4 x 70 mm^2^) was used for fluid flow transfer to the detection zone. Stencil printing of fluidic channels is illustrated in **Figure S1**.


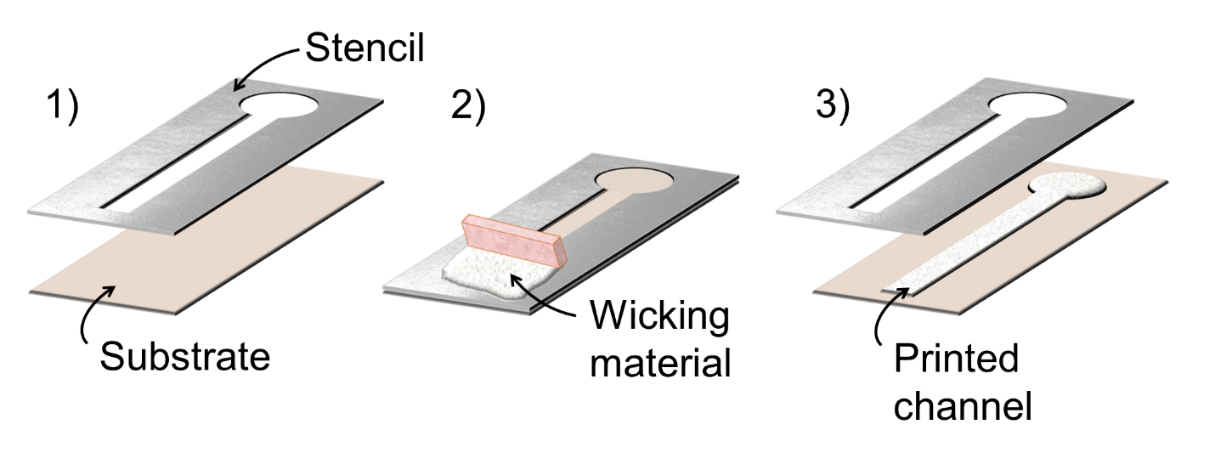


**Figure S1.** Schematic illustration of stencil printing of fluidic channels on paper support.

**Scanning Electron Microscopy (SEM).** The printed fluidic channels and the corresponding thin model films deposited on QCM-D crystals were imaged using an SEM. Before imaging, all the samples were sputter-coated with a 4-nm Au-Pd layer using a LEICA EM ACE600 sputter coater. Images of the channels were taken with a field emission microscope (Zeiss Sigma VP) operated at 1.5 kV. Each sample was imaged at least from three different spots.

**QCM data analysis and mass estimations.** The adsorbed mass was calculated following the Sauerbrey equation:

$\Delta m=-C_{QCM}\frac{\Delta f}{n}$,

where *C_QCM_* is 17.7 ng/(Hz×cm^2^) for a 5 MHz crystal, Δ𝑓 is the change in frequency, and *n* is the overtone number(Sauerbrey 1959; Rodahl and Kasemo 1996). In addition, Voigt viscoelastic modeling (Q‐Tools software, version 2.1 Q‐Sense) was used to estimate the effect of viscoelastic property changes of the film on the adsorbed protein mass. In the model, the fluid density and viscosity were set to 1000 kg m^−3^ and 0.001 m^3^ kg^−1^, and the density was approximated to be 1300 kg m^−3^ for the adsorbed protein layer(Campbell and Kim 2007).

**Fluorescein labeling of hIgG.** Antibodies were modified with a fluorescent probe by using the procedure of Hermanson(Hermanson 2008) with few alterations. First, 2 mg/mL protein solution was prepared in 0.1 M sodium carbonate buffer (pH 9.0). Then, 1 g/L FITC solution was prepared in a dark room by dissolving FITC in dry DMSO. This solution was protected from light by wrapping the bottle using aluminum foil. Next, 100 μL of the FITC solution was added dropwise to each mL of hIgG solution and gently mixed. The reaction occurred at 4 °C for 8 h. To purify the obtained FITC-stained hIgG (hIgG-FITC) from unreacted FITC molecules, the solution was centrifuged four times at 4000 rpm for 30 min by using centrifugal filter units (Amicon Ultra-15, MWCO 30 kDa).

**Adsorption capabilities of printed NPcat and PEI patterns.** *Dye adsorption test*: The adsorption capabilities of printed NPcat and PEI patterns were studied with a fluorescein-based dye. First, 0.05 wt% dye solution was dropped on the patterned substrates with a pipette. Then, the substrates were washed with MilliQ water. The formation of the patterns was evaluated by visual observation. Each sample was tested at least three times. *Protein interactions and confocal imaging*: We studied protein adsorption onto NPcat- and PEI-patterns printed on nanopapers. These films were prepared from cellulose nanofibrils (CNF) and were thin enough to permit the detection of the patterns with confocal microscopy. Specifically, 10 µL of 0.1 mg/mL hIgG-FITC (in phosphate buffer, pH 7.4) was adsorbed on the films with NPcat and PEI print patterns. Then, washing was done with the buffer. Next, the samples were imaged with CLSM by using 633 V laser power and constant imaging conditions. Additionally, specific protein interactions were studied using similar methods. First, 10 µL of 0.1 mg/mL anti-hIgG was adsorbed on the printed patterns and washed. Then, 10 µL of 0.5 wt% BSA was added and washed. Finally, 10 µL of 0.1 mg/mL hIgG-FITC was introduced, and after washing, the samples were imaged with CLSM. Notably, at least two parallel samples were imaged per sample.


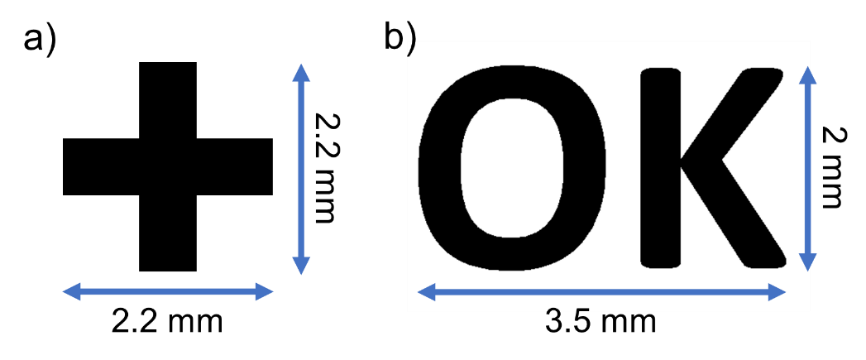


**Figure S2.** Printed patterns: a) Positive sign corresponds to the test line of an immunoassay and is used for the deposition of the capture antibodies (SARS-CoV-2 nucleocapsid antibody). b) “OK” sign corresponds to the control line, this pattern is either left untouched or secondary antibodies (anti-mouse IgG) are deposited on top.


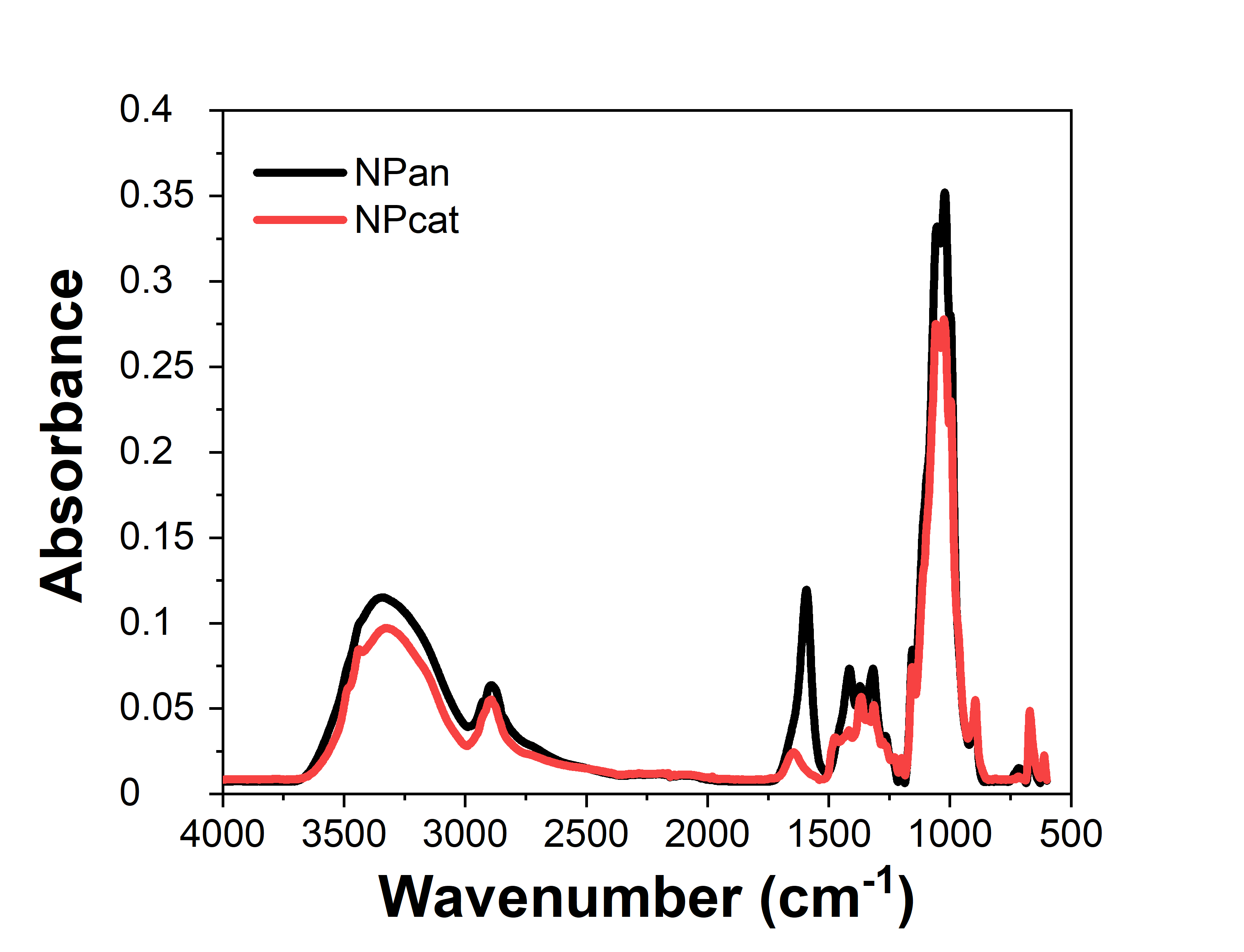


**Figure S3.** FTIR spectra of freeze-dried NPan and NPcat samples. The appearance of a carbonyl band (approx. 1610 cm^-1^) in the spectrum of NPan verifies the carboxymethylation. The C-N band (approx. 1510 cm^-1^) in the spectrum of NPcat corresponds to the introduced cationic trimethylammonium moiety proving the successful cationization.

**Table S1.** Colloidal properties of the NPan and NPcat (0.05 wt% in 2.5 mM NaCl buffer).

| Sample | Zeta-potential (mV) | Electrophoretic mobility (µmcm/Vs) | Number-averaged particle size (nm) |
| --- | --- | --- | --- |
| NPan | -25 | -1.9 | 65 |
| NPcat | 20 | 1.6 | 58 |

**
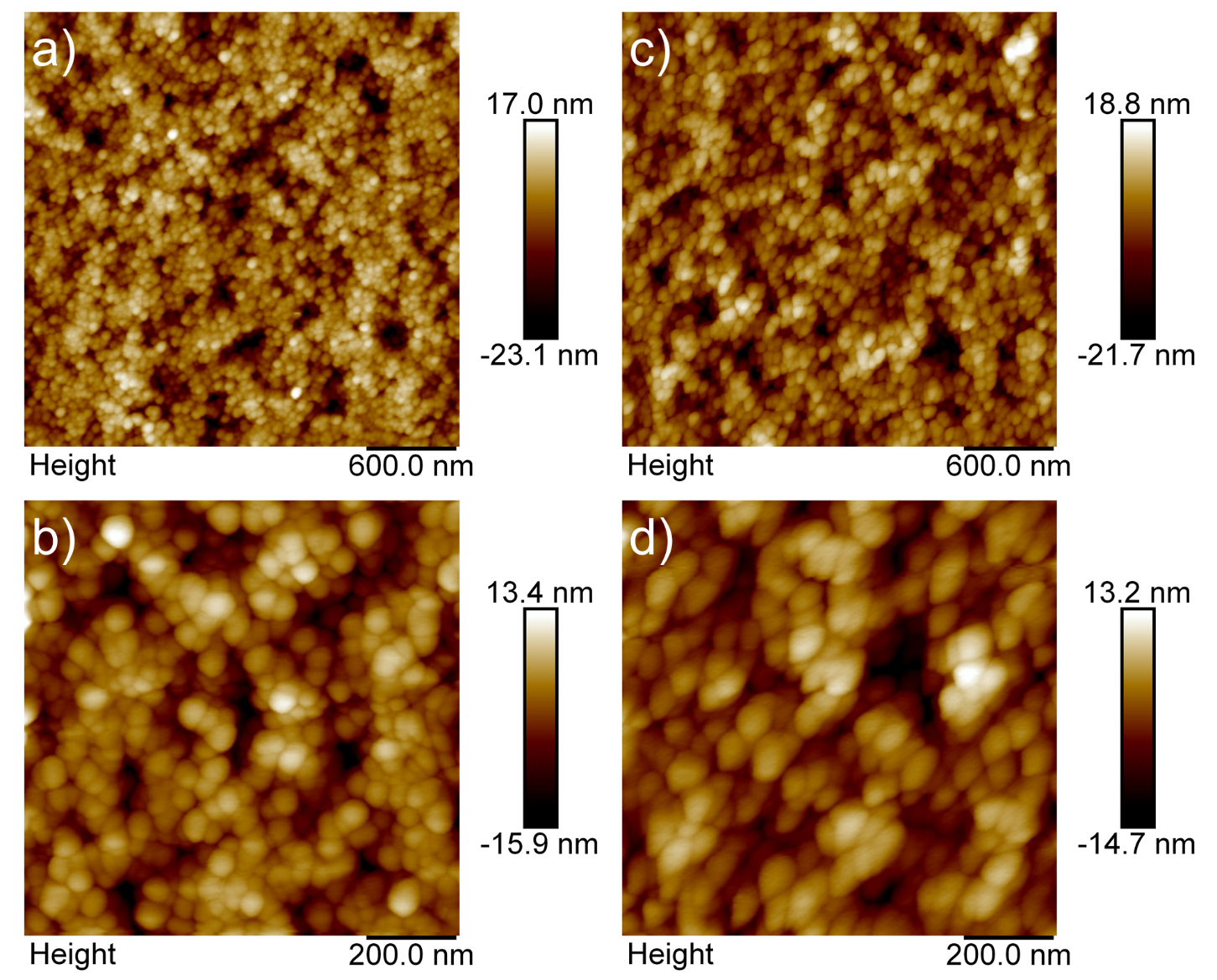
**

**Figure S4.** AFM images of a-b) NPan and c-d) NPcat films on a silicon wafer. In the NPcat images, the deformation of nanoparticles is most likely caused by the attraction of the tip and the cationic particle.


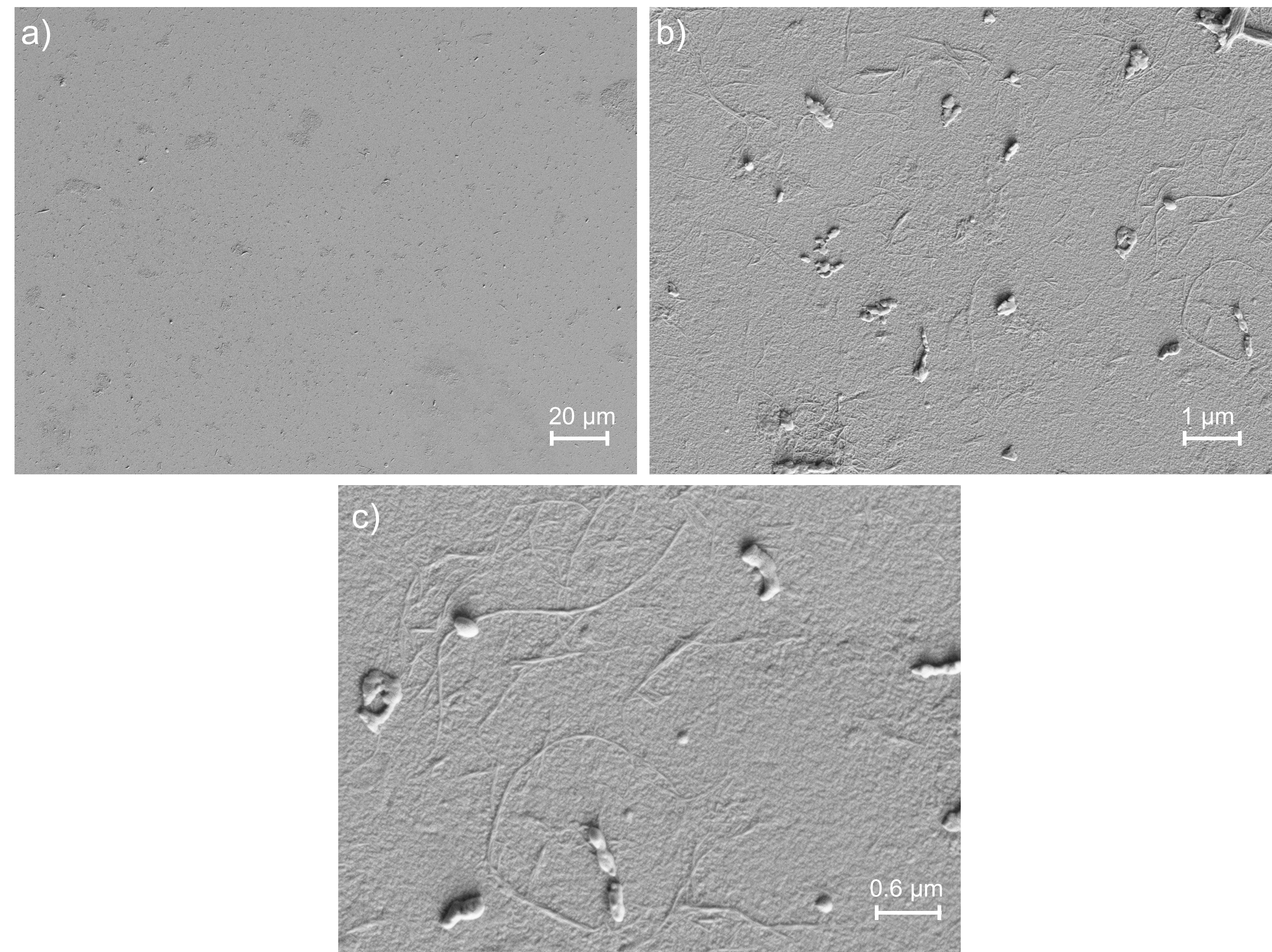


**Figure S5.** SEM images of the ultrathin films of the CaCH paste on QCM-D crystals: a) magnification $\times$500, b) magnification $\times$10 000, and c) magnification $\times$20 000.


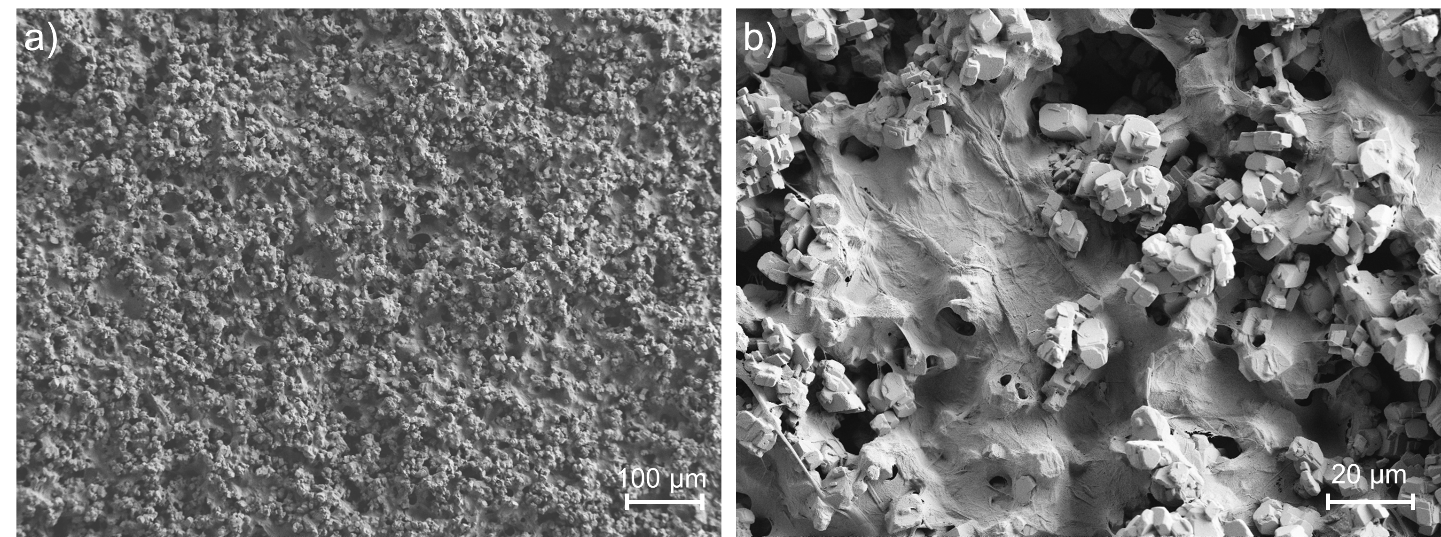


**Figure S6.** SEM images of the printed fluidic channels comprising CaCO_3_, HefCel, CNF, and propylene glycol (Ca-CH) showing the porous surface structure: a) magnification $\times$100, and b) magnification $\times$700.


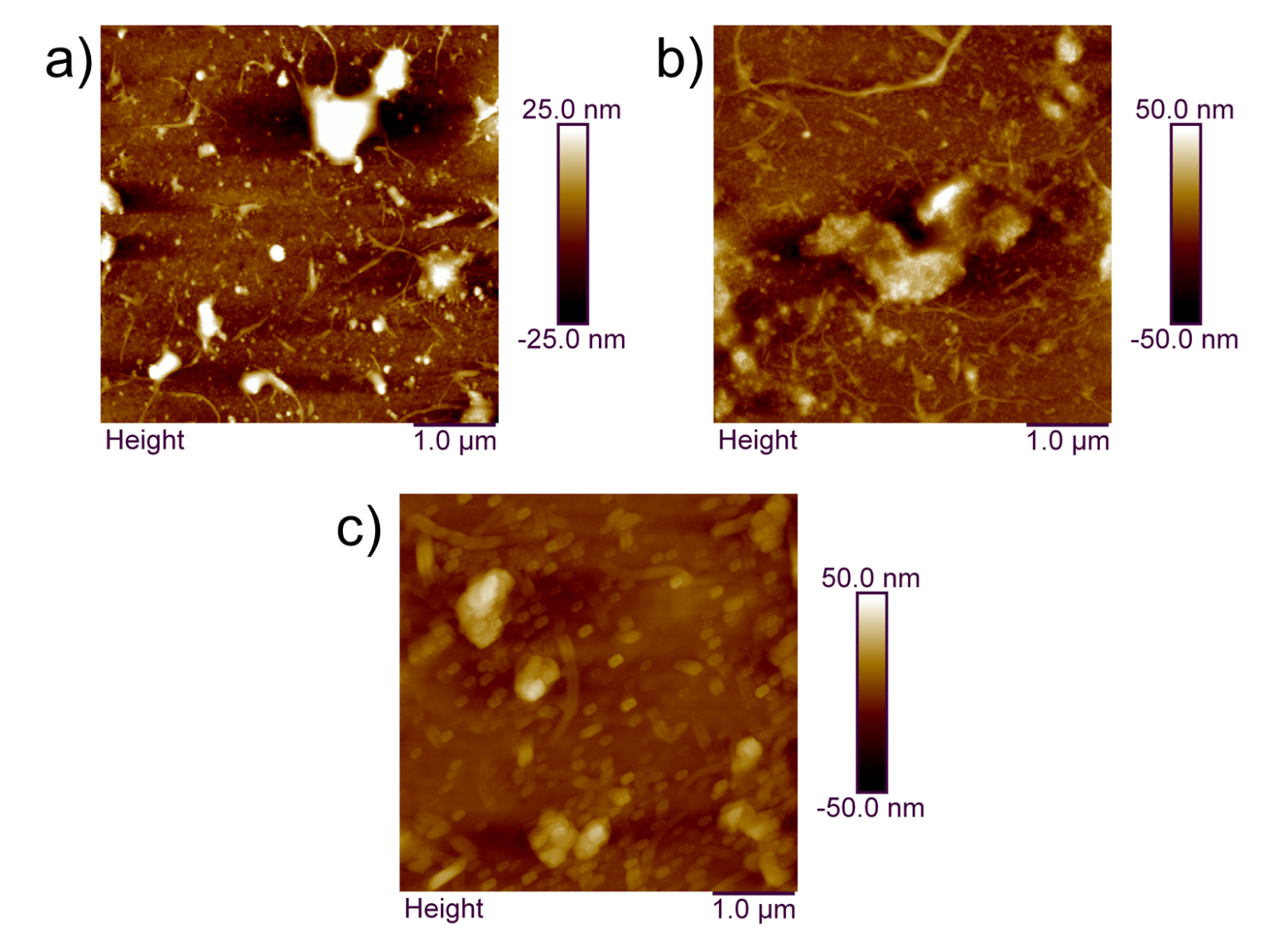


**Figure S7.** AFM images of the QCM-D crystals: a) unmodified model surface, b) NPcat modified model surface, and c) PEI modified model surface (image distorted most likely due to attraction of the surface and the tip).

**Table S2.** Measured energy dissipation changes and calculated masses of the adsorbed BSA and fibrinogen on each model surface.

| Adsorption of BSA | | | |
| --- | --- | --- | --- |
| Surface | **Δ*D* (x10^-6^)** | **Δ*m*_Voigt_ (ng/cm^2^)** | **Δ*m*_Sauerbrey_ (ng/cm^2^)** |
| Unmodified | 1.5 | 637 | 576 |
| PEI | 0.6 | 515 | 571 |
| NPcat | 5.3 | 1345 | 981 |
| NPan | -0.1 | 14 | 23.8 |
| Adsorption of fibrinogen | | | |
| Surface | **Δ*D* (x10^-6^)** | **Δ*m*_Voigt_ (ng/cm^2^)** | **Δ*m*_Sauerbrey_ (ng/cm^2^)** |
| Unmodified | 0.2 | 188 | 176 |
| PEI | 0.1 | 205 | 232 |
| NPcat | 9.5 | 858 | 896 |
| NPan | -0.9 | 18 | 19.9 |
| BSA | 0.3 | 137 | 113 |


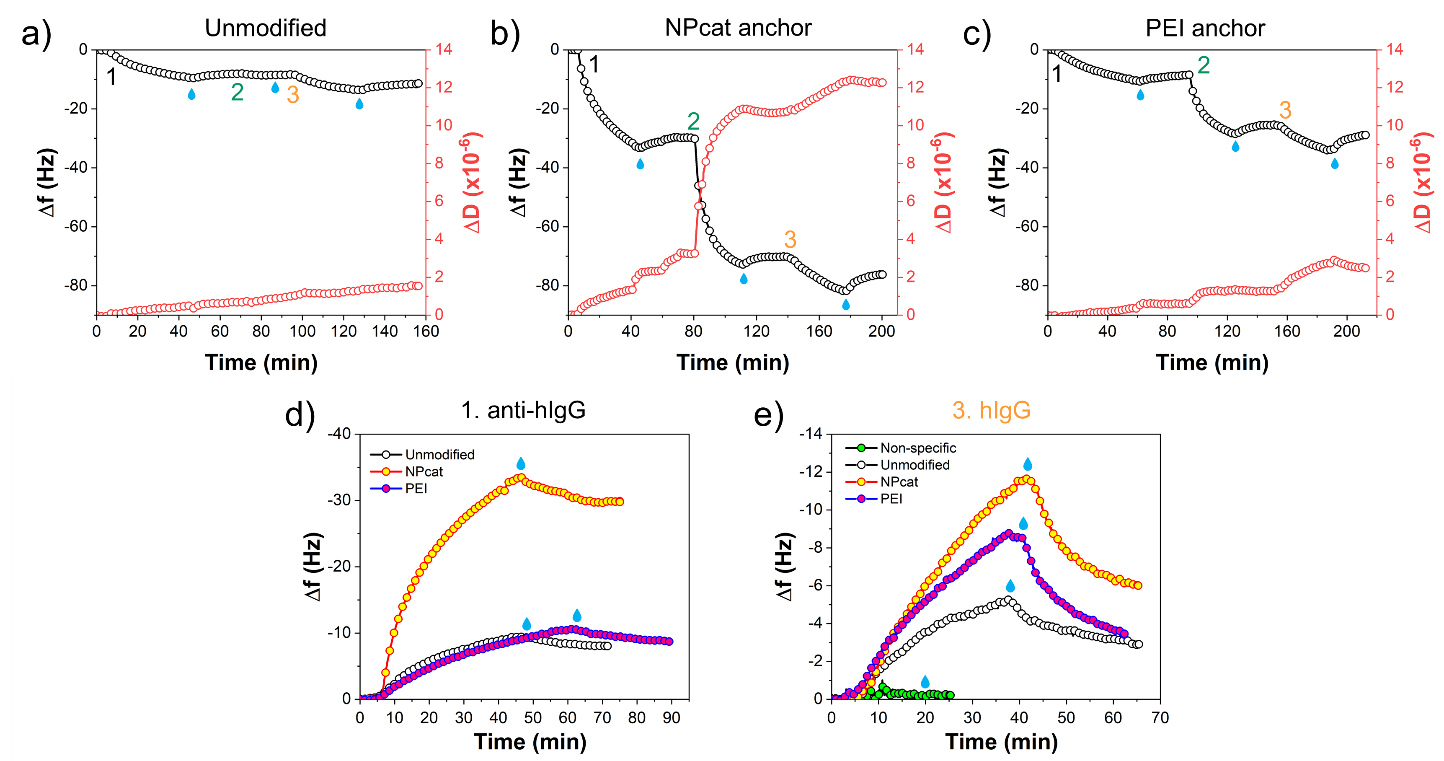


**Figure S8.** QCM-D data showing adsorption of anti-human IgG, BSA, and hIgG on a) unmodified model film and b) NPcat- and c) PEI-treated films. d) The effect of treatments on anti-hIgG adsorption. e) The effect of treatments on the specific adsorption of human IgG on anti-human IgG modified surfaces after BSA blocking, non-specific adsorption of hIgG on BSA-blocked model film is given as a comparison. The blue droplet indicates rinsing with buffer.


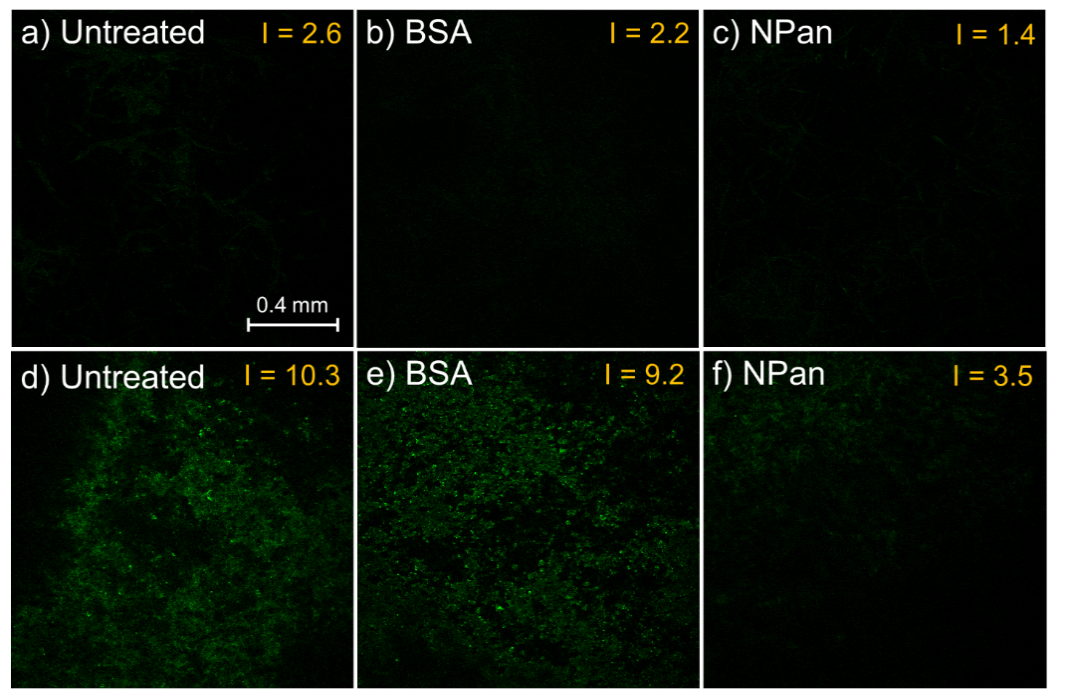


**Figure S9.** Confocal images of filter papers and printed fluidic channels exposed to fluorescent-labeled protein (hIgG-FITC) and effect of blockers: a) unmodified filter paper, b) BSA-treated filter paper, c) NPan-treated filter paper, d) unmodified printed channel, e) BSA-treated channel and f) NPan-treated channel. The intensity of fluorescence is indicated in each sample. Images were taken with 750 V laser power.


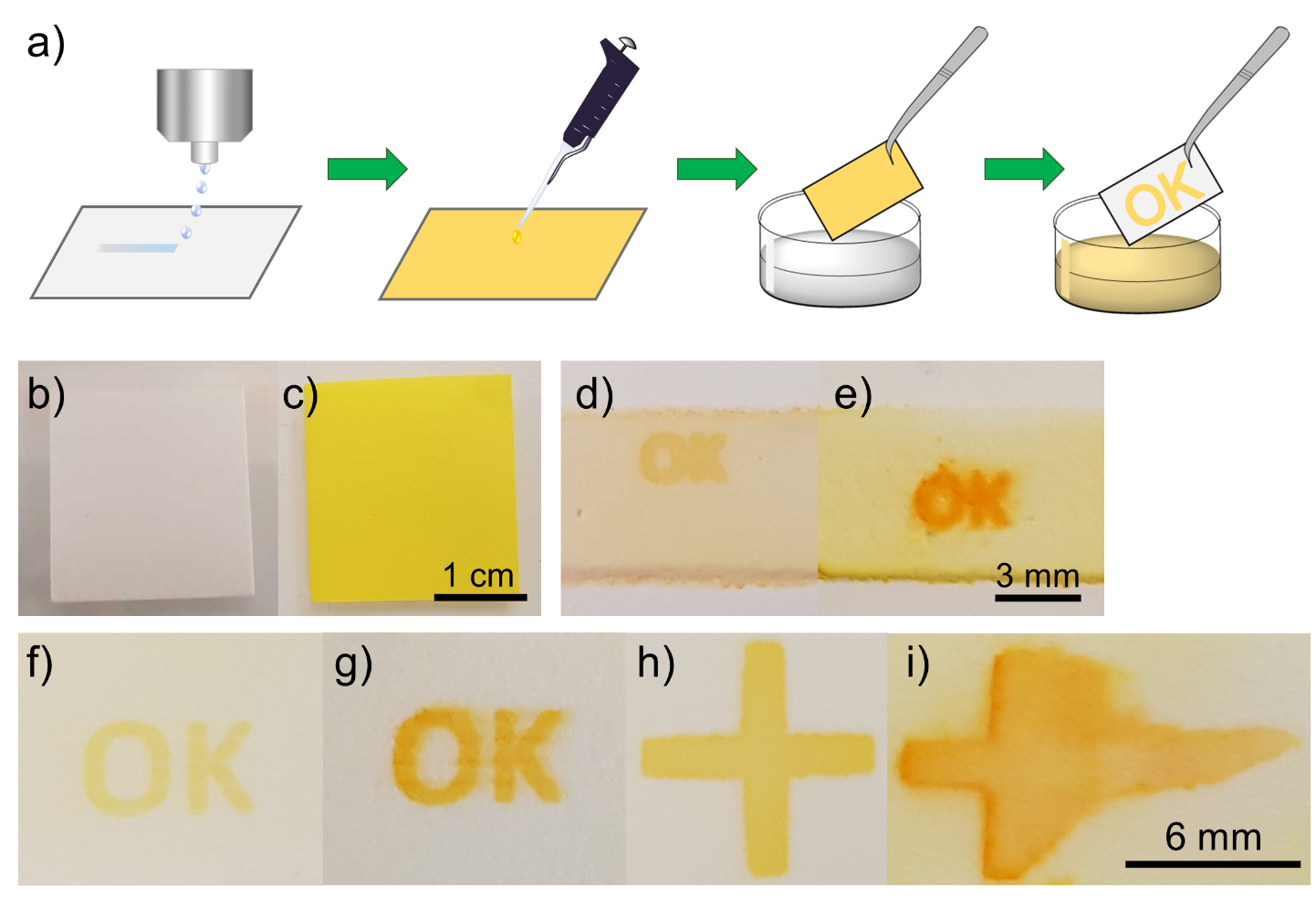


**Figure S10.** A *proof-of-concept* test illustrating the immobilization capability of cationic materials demonstrated by the adsorption of a fluorescein-based dye. a) Schematic illustration of the test system: First NPcat or PEI was inkjet-printed to form a pattern (invisible) on filter paper. Next, the sample was exposed to a dye solution. Finally, the substrate was washed with MilliQ to reveal the patterns. Images of b) filter paper with printed NPcat pattern (invisible) and b) fluorescein-dyed filter paper (with the NPcat pattern). Images of the washed samples showing high adsorption capability of NPcat and PEI on the printed fluidic channel: d) NPcat pattern and c) PEI pattern (1 print layer). f-i) Images of the washed samples showing high adsorption capability of NPcat and PEI on filter paper. Patterns with one layer: f) NPcat, and g) PEI. Patterns with five layers: h) NPcat, and i) PEI. NPcat formed clear patterns with sharp edges, whereas the PEI patterns were somewhat distorted by fluid transport/flow.


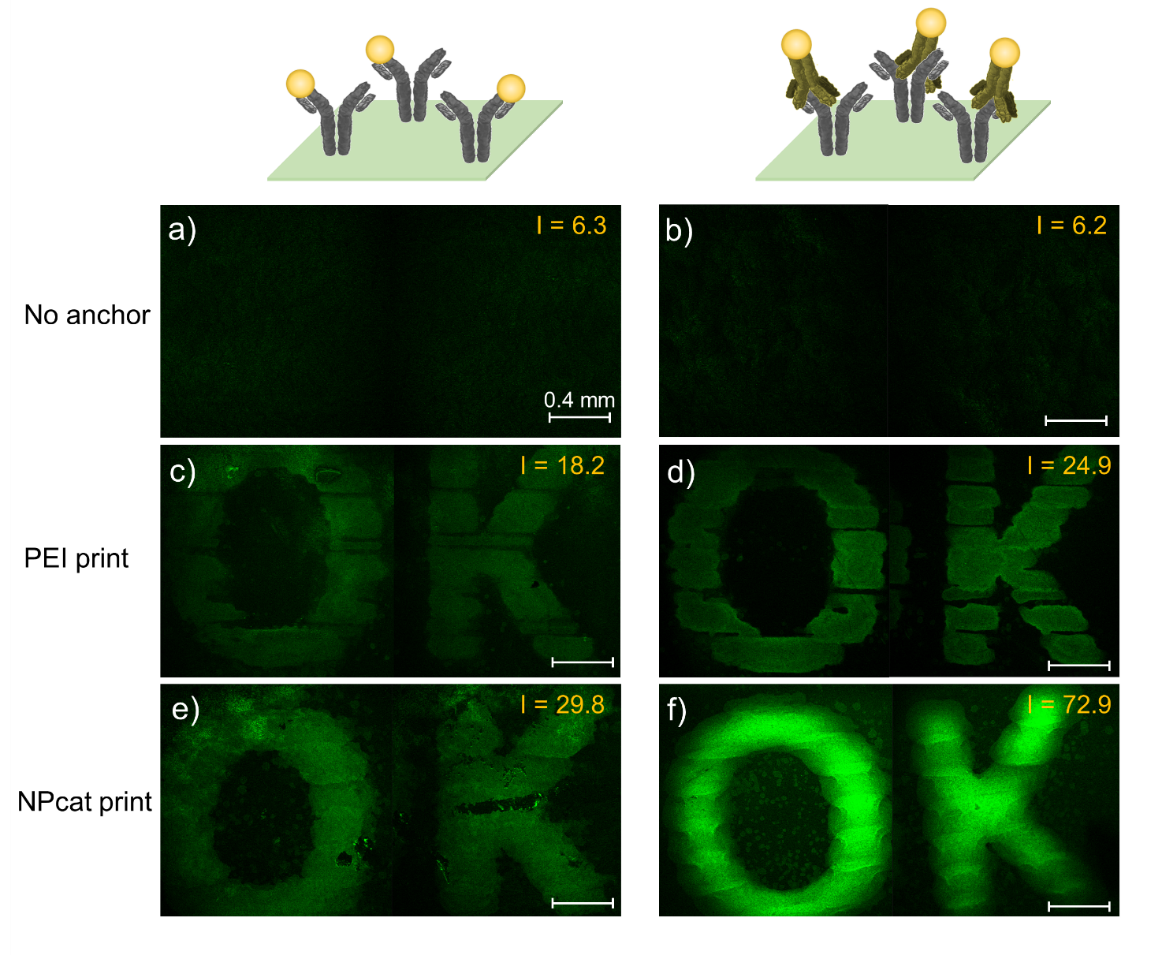


**Figure S11.** Confocal images showing non-specific (left) and specific (right) protein adsorption onto a-b) unmodified nanopaper, and nanopapers printed with c-d) PEI and e-f) NPcat patterns. In non-specific interactions, hIgG-FITC was adsorbed directly on the samples. For specific interactions, hIgG-FITC was adsorbed on anti-hIgG-treated samples. Imaging was done after washing samples with a buffer solution. The fluorescence intensity is indicated in each image. Scale bar in each image 0.4 mm.


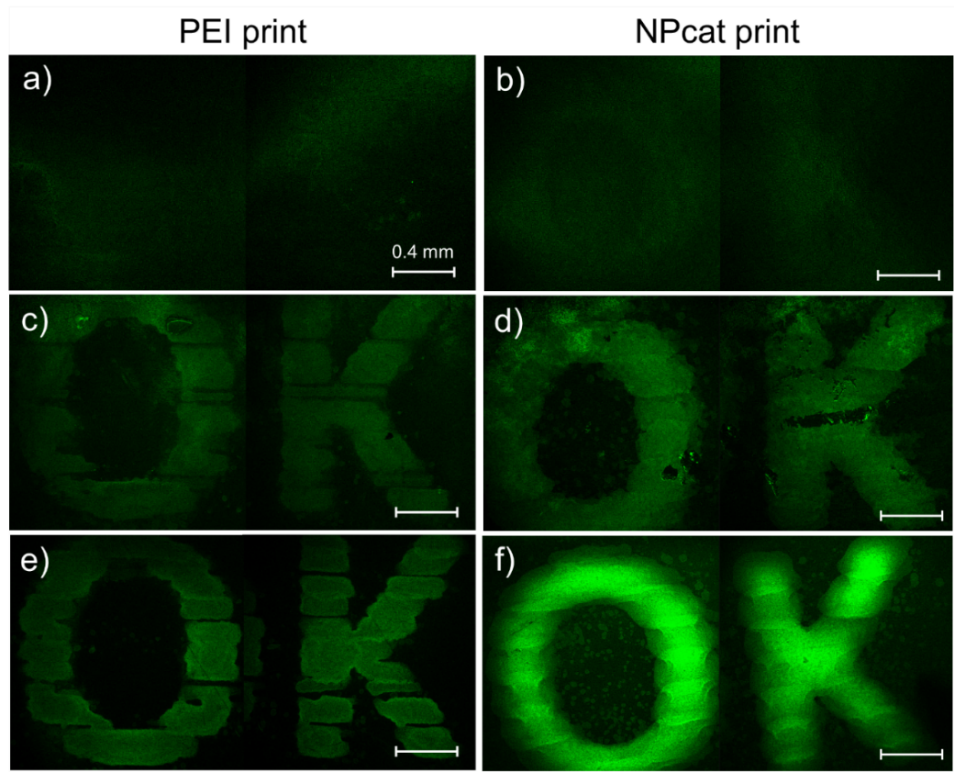


**Figure S12.** Confocal images of NPcat and PEI patterns printed on nanopapers showing adsorption of fluorescent-labeled proteins. a) PEI pattern (without proteins), b) NPcat pattern (without proteins), c) PEI pattern with non-specifically adsorbed hIgG-FITC, d) NPcat pattern with non-specifically adsorbed hIgG-FITC. Images were taken with 630 V laser power.


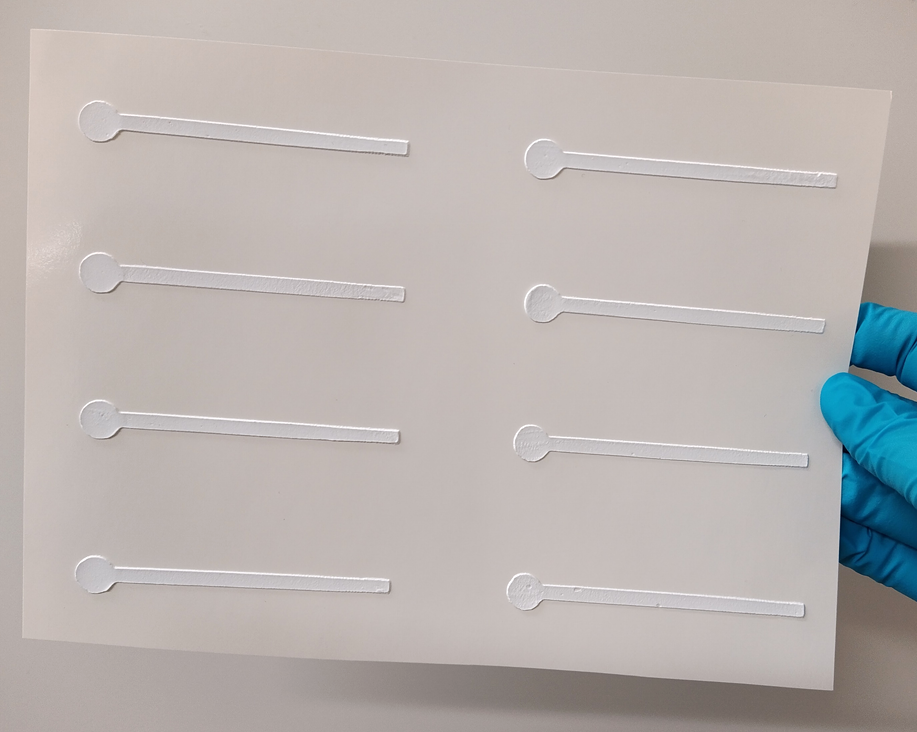


**Figure S13.** Printed fluidic channels on PowerCoat HD^®^ paper substrate. Channels consist of an initial circular section for sample insertion and a rectangular channel for fluid transportation. These channels were used for the preparation of the immunoassays. The fluidic channels printed on PowerCoat^®^ paper showed repeatable designs obtained by stencil printing. The fluid wicking properties of these channels have been analyzed in our previous work, where we reported that the channels wicked 4 cm of water in approx. 130 s(Solin et al. 2021).


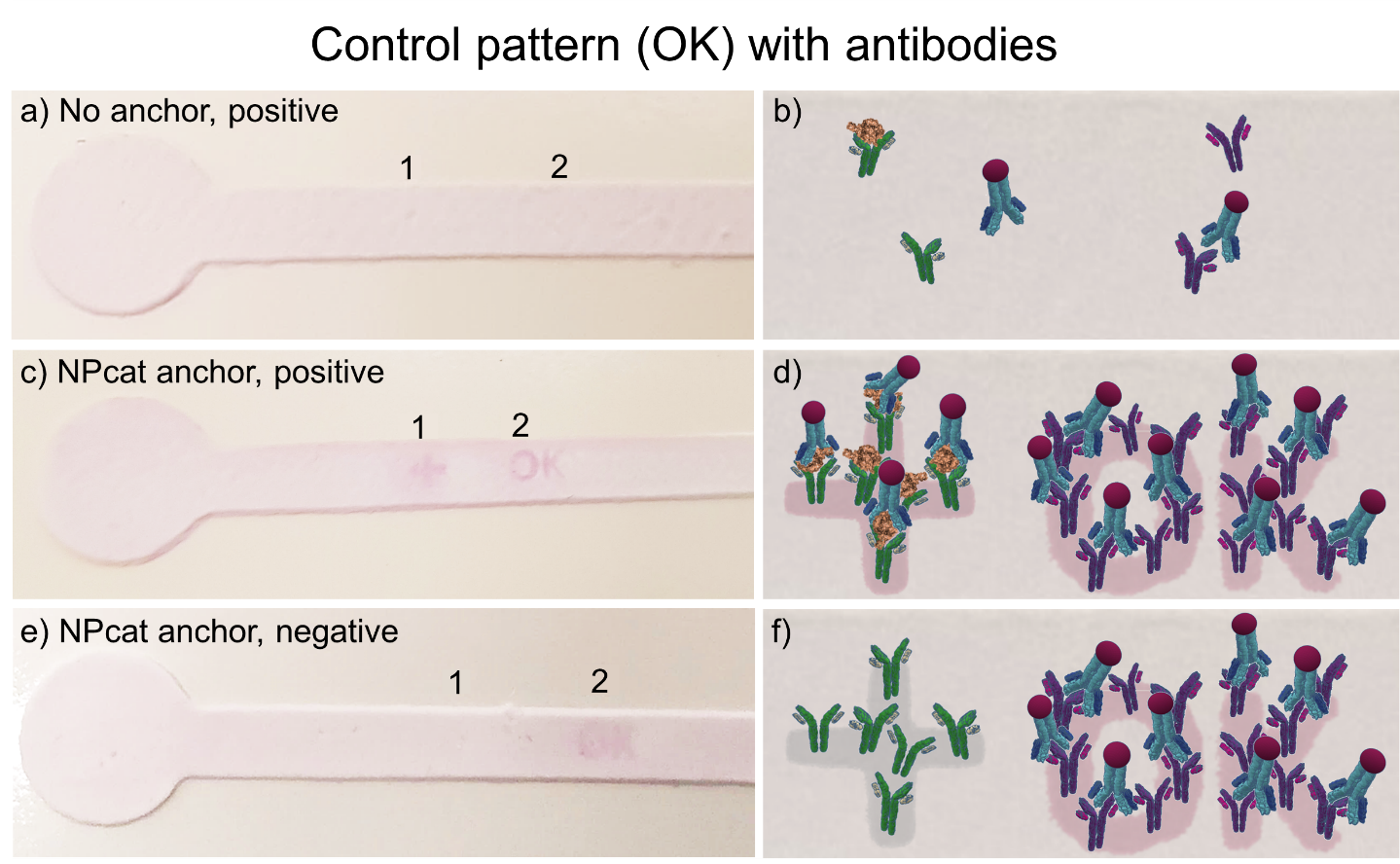


**Figure S14.** Effect of NPcat anchor layer on detection, antibodies on both test and control pattern. No signal was detected on assay a-b) without anchor layer (capture antibody deposited on the test area, secondary antibody deposited on control area, positive sample tested). Immobilization of the sensing elements with NPcat anchor: c) image of an assay tested with a positive sample (8 ng/mL N protein) and d) schematic illustration showing increased adsorption of detection antibody on capture antibody-nucleocapsid complex (test area) and secondary antibody (control area), e) image of channel tested with a negative sample and f) schematic illustration demonstrating adsorption of detection antibody on to the secondary antibody -covered control area.


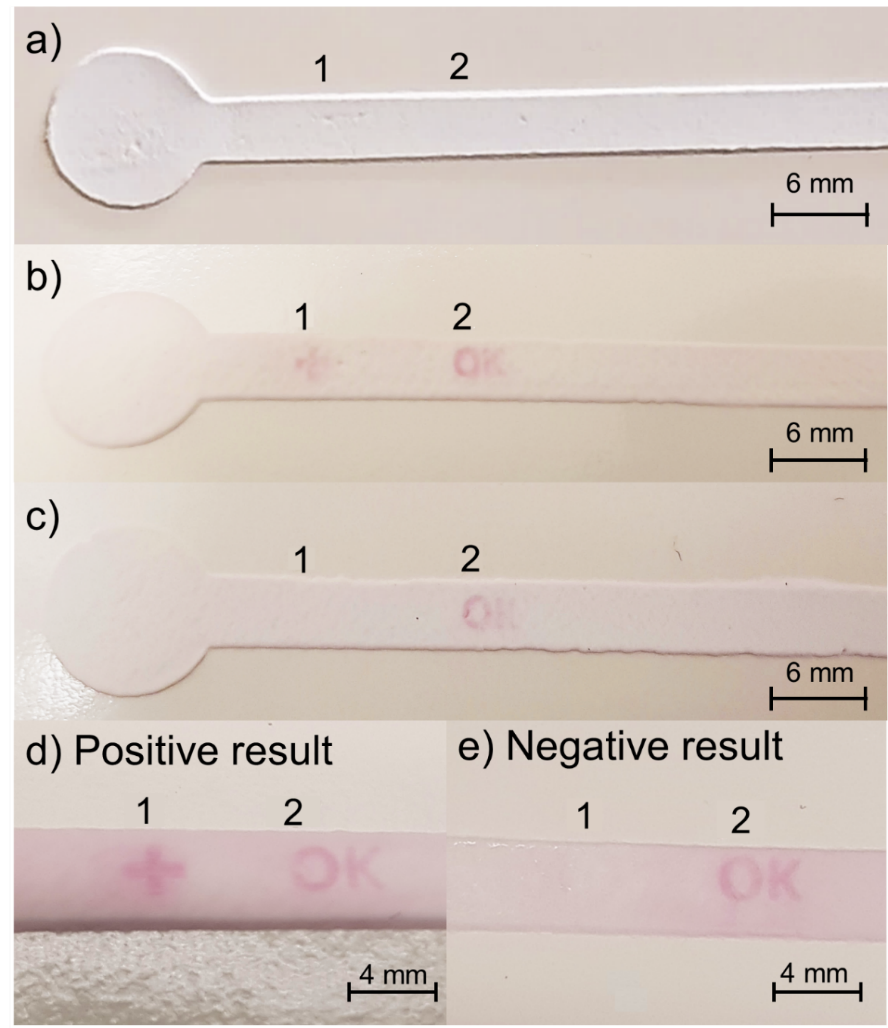


**Figure S15.** Images of the prepared immunoassays with paper background showing colorimetric responses before and after testing: a) untested assay with marked positions of the test (1) and control (2) areas (patterns invisible), tested and washed assays exposed to b) antigen-positive sample (8 ng/mL N protein) and c) antigen-negative sample. Corresponding color development in unwashed assays: d) antigen-positive sample and e) antigen-negative sample.


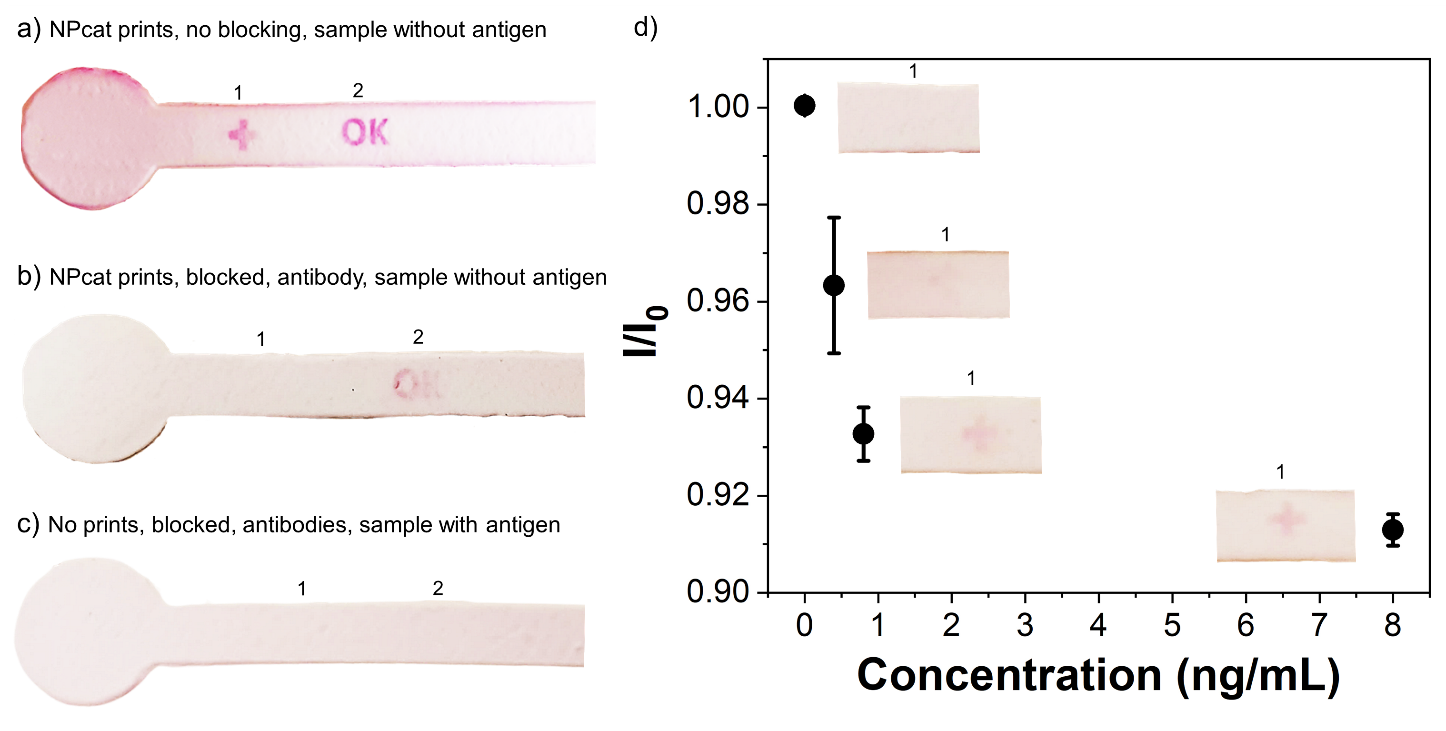


**Figure S16.** Effect of anchor layer, blocking treatments, and sample concentration on the performance of the immunoassay: a) A false-positive result and significant background adsorption on an untreated fluidic channel, with only printed NPcat patterns (without NPan blocking, no antibodies or other proteins) upon testing with antigen-negative sample. b) Immunoassay prepared on the NPan-treated fluidic channel with deposited capture antibodies on the test area (with BSA blocking) produced a clear negative result upon testing with antigen-negative sample. c) No detectable signal developed in the absence of NPcat anchor patterns upon testing with antigen-positive sample (with 8 ng/mL N protein) on the NPan-blocked assay with capture (1) and secondary (2) antibodies. d) Normalized intensity of coloration on the testing zone as a function of N-protein concentration (0, 0.4, 0.8 and 8 ng/mL) revealed visual detection limit of approx. 0.4 ng/mL, which caused a faint-colored positive sign on the test zone. I/I_0_ was obtained by dividing the color intensity on the test zone with the color intensity of the background channel. Decrease in intensity indicates darker coloration on the test pattern.


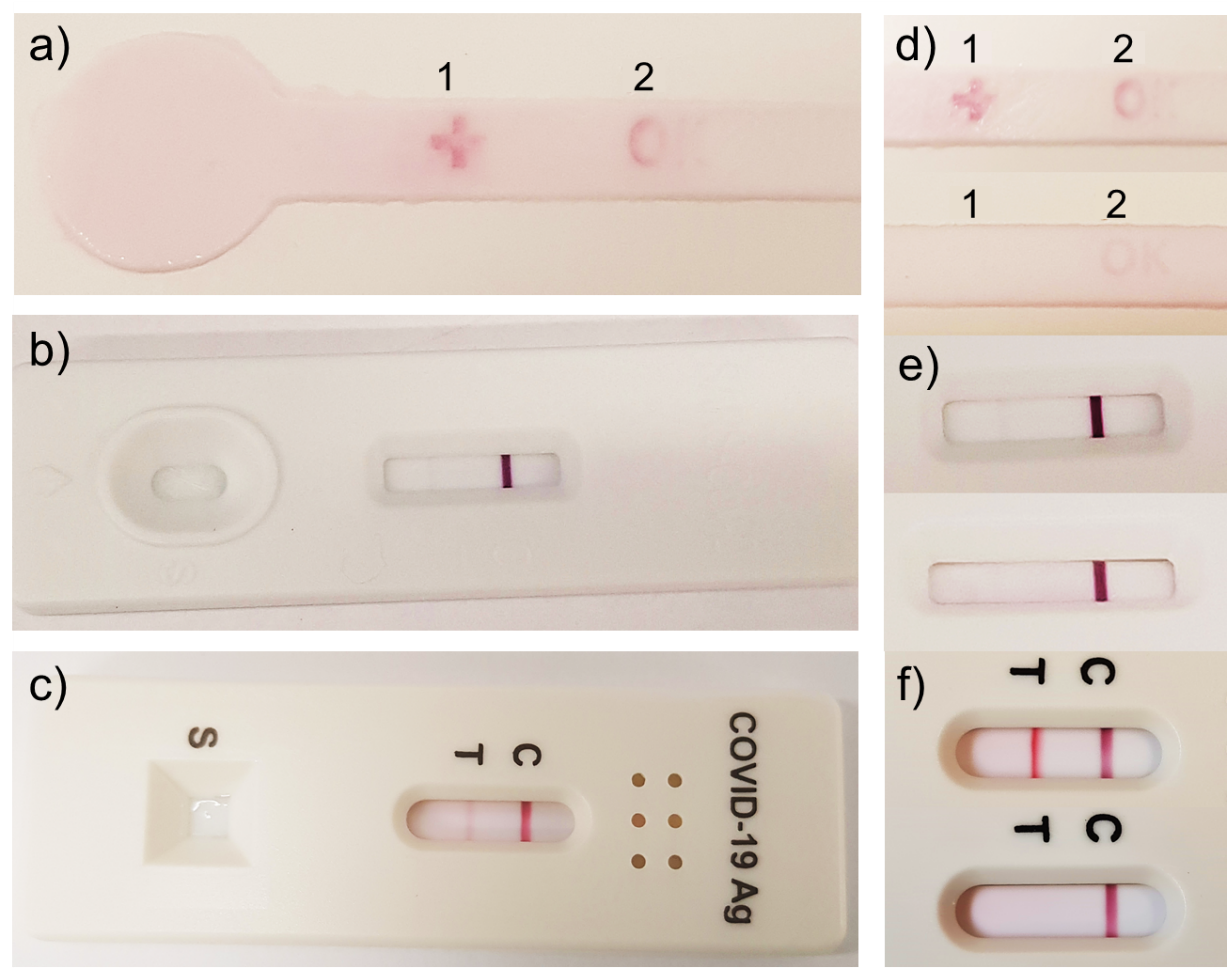


**Figure S17.** a-c) Testing positive saliva samples on our developed assay and comparison with commercial detection devices. The test was performed by mixing saliva and the provided buffer solution in a 1:1 ratio. To obtain a positive sample, N protein was mixed to the diluted saliva sample to gain 1 µg/mL final concentration. Clear positive results were obtained with a) our developed assay in 3 min (three parallel samples, no washing), b) an exemplary commercial assay (Device A) showed only one faded positive test line in 10 min and lacked reproducibility (tested three times; one faded positive and two false-negative results), c) the other exemplary commercial test (Device B) produced positive results in 2-9 min (three parallel samples, the intensity of the test line color and test time varied). Negative saliva samples were also tested and compared with the positive results: Zoomed images of the sensing areas of d) the developed assay, e) Device A and f) Device B tested with positive (above) and negative (below) saliva samples. Device B and the developed assay showed good performance.

**References:**

Beaumont M, Kondor A, Plappert S, et al (2017) Surface properties and porosity of highly porous, nanostructured cellulose II particles. Cellulose 24:. https://doi.org/10.1007/s10570-016-1091-y

Beaumont M, Nypelö T, König J, et al (2016a) Synthesis of redispersible spherical cellulose II nanoparticles decorated with carboxylate groups. Green Chemistry 18:1465–1468. https://doi.org/10.1039/c5gc03031e

Beaumont M, Rennhofer H, Opietnik M, et al (2016b) Nanostructured Cellulose II Gel Consisting of Spherical Particles. ACS Sustain Chem Eng 4:4424–4432. https://doi.org/10.1021/acssuschemeng.6b01036

Beaumont M, Rosenfeldt S, Tardy BL, et al (2019) Soft cellulose II nanospheres: Sol-gel behaviour, swelling and material synthesis. Nanoscale 11:17773–17781. https://doi.org/10.1039/c9nr05309c

Bussmann BM, Reiche S, Jacob LH, et al (2006) Antigenic and cellular localisation analysis of the severe acute respiratory syndrome coronavirus nucleocapsid protein using monoclonal antibodies. Virus Res 122:119–126. https://doi.org/10.1016/J.VIRUSRES.2006.07.005

Campbell CT, Kim G (2007) SPR microscopy and its applications to high-throughput analyses of biomolecular binding events and their kinetics. Biomaterials 28:2380–2392

Hermanson GT (2008) Bioconjugate techniques, 2nd editio. Academic Press, New York, USA

Mertens P, de Vos N, Martiny D, et al (2020) Development and Potential Usefulness of the COVID-19 Ag Respi-Strip Diagnostic Assay in a Pandemic Context. Front Med (Lausanne) 7:225. https://doi.org/10.3389/fmed.2020.00225

Pere J, Tammelin T, Niemi P, et al (2020) Production of High Solid Nanocellulose by Enzyme-Aided Fibrillation Coupled with Mild Mechanical Treatment. ACS Sustain Chem Eng 8:18853–18863. https://doi.org/10.1021/acssuschemeng.0c05202

Rodahl M, Kasemo B (1996) On the measurement of thin liquid overlayers with the quartz-crystal microbalance. Sens Actuators A Phys 54:448–456. https://doi.org/10.1016/S0924-4247(97)80002-7

Sauerbrey G (1959) Verwendung von Schwingquarzen zur Wägung dünner Schichten und zur Mikrowägung. Zeitschrift für Physik 155:206–222. https://doi.org/10.1007/BF01337937

Solin K, Beaumont M, Rosenfeldt S, et al (2020) Self‐Assembly of Soft Cellulose Nanospheres into Colloidal Gel Layers with Enhanced Protein Adsorption Capability for Next‐Generation Immunoassays. Small 2004702. https://doi.org/10.1002/smll.202004702

Solin K, Borghei M, Imani M, et al (2021) Bicomponent Cellulose Fibrils and Minerals Afford Wicking Channels Stencil-Printed on Paper for Rapid and Reliable Fluidic Platforms. ACS Appl Polym Mater 3:5536–5546. https://doi.org/10.1021/acsapm.1c00856

Tammelin T, Hippi U, Salminen A (2013) Method for the preparation of NFC films on supports

(2020) PowerCoat® HD - Arjowiggins Creative Papers. https://powercoatpaper.com/products/powercoat-hd/. Accessed 3 Apr 2020
